# Machine Learning Prediction of Litter Moisture and Litter pH in a Commercial Broiler House Using Nested Leave-One Production-Period-Out Cross-Validation

**DOI:** 10.3390/ani16182906

**Published:** 2026-09-15

**Authors:** Erdem Küçüktopçu, Bilal Cemek

**Affiliations:** Department of Agricultural Structures and Irrigation, Ondokuz Mayıs University, Samsun 55139, Türkiye; erdem.kucuktopcu@omu.edu.tr

**Keywords:** litter moisture, litter pH, machine learning, precision livestock farming, environmental monitoring, poultry production

## Abstract

Litter moisture content (LMC) and litter pH (LPH) are important indicators of litter quality in broiler production. This study developed machine-learning models to predict LMC and LPH using environmental, flock-related, spatial, and litter-related variables collected from eight temporally distinct production periods in the same commercial broiler house at days 7, 21, and 42 (1600 observations). Four ML algorithms, namely Support Vector Regression (SVR), Random Forest (RF), Extreme Gradient Boosting (XGB), and Multilayer Perceptron (MLP), were evaluated under six predictor scenarios (S1–S6) using nested Leave-One-Production-Period-Out cross-validation. For LMC, XGB under S3 achieved the highest mean outer-fold R^2^ (0.796), XGB under S5 the lowest RMSE (3.023) and MAPE (8.356%), and SVR under the litter-assisted S6 scenario the lowest MAE (2.433). For LPH, RF showed consistently competitive performance and achieved R^2^ = 0.891, RMSE = 0.181, MAE = 0.139, and MAPE = 1.922% under the litter-assisted S6 scenario. These comparisons were interpreted as exploratory, metric-specific benchmarking rather than definitive model selection. The nested framework provided grouped internal validation across held-out production periods within the studied house. Because all periods originated from one house, broader generalization requires validation in additional commercial facilities. Prospective validation is also required before operational deployment or assessment of management, welfare, environmental, or sustainability benefits.

## Simple Summary

Poultry litter conditions can change considerably during a broiler production cycle, while frequent laboratory measurement of litter moisture and pH can be labor-intensive. This study investigated whether machine-learning methods could estimate these two important litter characteristics using information on the broiler-house environment, bird growth stage, sampling location, and litter conditions. Data were collected during eight production periods in the same commercial broiler house. To provide a more realistic assessment, each production period was excluded from model development in turn and then used to evaluate prediction performance. The results show that litter moisture and litter pH could be estimated with useful predictive accuracy, although performance varied according to the information included in the models. Because all data were obtained from a single broiler house, the findings should be considered specific to the conditions represented in this study. The proposed approach may provide a basis for future litter-monitoring and decision-support systems, but further validation in different commercial broiler houses is required before practical application.

## 1. Introduction

The rapid expansion of the poultry industry has intensified the need for precision environmental management to ensure sustainable production, animal welfare, and economic efficiency [1]. Modern broiler production systems operate under high stocking densities where even minor deviations in environmental conditions can substantially influence bird health, productivity, litter quality, and gaseous emissions. Consequently, continuous monitoring and accurate prediction of environmental indicators have become essential components of precision livestock farming, enabling producers to optimize management strategies while minimizing production losses and environmental impacts [2,3].

Among the numerous environmental indicators monitored in broiler houses, litter quality is one of the most critical determinants of housing conditions [4]. Litter functions not only as a bedding material but also as a dynamic biological substrate whose physical and chemical properties continuously change throughout the production cycle. Deterioration of litter quality directly affects bird welfare by increasing the incidence of footpad dermatitis, hock burns, breast blisters, respiratory diseases, and microbial infections. Furthermore, poor litter conditions accelerate ammonia volatilization, reduce indoor air quality, and increase environmental emissions, thereby affecting both animal performance and occupational health [5].

Litter moisture content (LMC) and litter pH (LPH) are widely recognized as the primary indicators of litter quality because they govern microbial activity, nutrient transformations, ammonia production, and pathogen survival within the litter matrix [6,7]. Their evolution is controlled by a complex interaction among environmental conditions, ventilation efficiency, bird growth, manure accumulation, litter temperature, spatial heterogeneity, and seasonal climatic variations [8]. These interacting processes are highly nonlinear and exhibit substantial temporal and spatial variability throughout successive production periods, making accurate prediction particularly challenging using conventional statistical techniques.

Traditional regression models generally rely on predefined functional relationships and assumptions of linearity, homoscedasticity, and independence among explanatory variables [9]. However, environmental processes occurring inside mechanically ventilated broiler houses rarely satisfy these assumptions because environmental variables interact simultaneously through complex biological and physical mechanisms [10]. Consequently, classical statistical approaches often fail to capture the nonlinear relationships governing litter dynamics, particularly under commercial production conditions where environmental variability is considerably greater than that observed in controlled experimental facilities.

Recent advances in machine learning (ML) have provided powerful alternatives for modeling complex agricultural systems. ML algorithms can capture nonlinear relationships directly from observed data without requiring explicit mathematical descriptions of the underlying processes. Accordingly, algorithms such as Support Vector Regression (SVR), Random Forest (RF), Extreme Gradient Boosting (XGB), and Multilayer Perceptron (MLP) have been applied to a wide range of precision livestock applications, including live weight estimation [11], thermal comfort assessment [12], disease detection [13], behavior recognition [14], and environmental monitoring [15]. Their ability to model nonlinear interactions has made them increasingly attractive for data-driven monitoring and decision-support applications in modern livestock production.

Despite these advances, the application of ML to litter quality prediction remains limited. Most previous studies have focused primarily on broiler growth performance, ammonia concentration, thermal comfort, ventilation control, or energy consumption, whereas predictive modeling of LMC and LPH has received considerably less attention. More importantly, existing studies frequently rely on conventional random train–test data partitioning strategies [15,16,17], allowing observations from the same production cycle to appear simultaneously in both the training and testing datasets. Such validation procedures can produce optimistic estimates of predictive performance because observations from a production period used during model development may share flock-specific, temporal, environmental, or management characteristics with observations included in the test subset. Spatial variability within commercial broiler houses and the contribution of different predictor groups are also relevant considerations in the development and evaluation of predictive models. Therefore, validation strategies that preserve production-period grouping are needed to provide a more stringent assessment of predictive performance on production periods excluded from model development.

Production periods in commercial broiler production may differ in environmental conditions, flock characteristics, management practices, and seasonal influences. When repeated observations from the same production period are randomly distributed between training and testing datasets, shared period-specific characteristics may lead to overly optimistic estimates of predictive performance. Group-based validation strategies can reduce this risk by ensuring that observations belonging to the same production cycle are kept together during model development and evaluation. In this context, Leave-One-Production-Period-Out cross-validation (LOPO-CV) provides a more stringent grouped assessment by evaluating models on complete production periods excluded from model development. However, when all production periods originate from the same commercial facility, such grouped validation represents within-house internal validation and should not be interpreted as external validation, which requires evaluation using independent facilities or populations not represented during model development.

Another important consideration is the pronounced spatial variability of environmental conditions within commercial broiler houses. Airflow distribution, heat production, bird activity, manure deposition, and moisture accumulation are inherently heterogeneous, resulting in significant spatial differences in litter characteristics [18,19]. Consequently, integrating environmental measurements with temporal information (bird age), seasonal conditions, and spatial coordinates has the potential to improve predictive performance while providing a more comprehensive representation of litter dynamics than environmental variables alone.

The main contributions of this study can be summarized as follows: (i) A nested LOPO-CV framework was applied to provide grouped internal validation across temporally distinct production periods, ensuring that all observations from each held-out production cycle were excluded from model development. (ii) Six progressively enriched predictor scenarios were evaluated to investigate the contributions of environmental, temporal, flock-related, spatial, and litter-related information. (iii) Four widely used ML algorithms (SVR, RF, XGB, and MLP) were evaluated under identical production-period-based validation conditions, with algorithm and scenario comparisons interpreted as exploratory benchmarking. (iv) The study characterizes predictor combinations associated with LMC and LPH predictive performance across held-out production periods within the studied commercial broiler house and provides a basis for subsequent validation in additional commercial facilities.

## 2. Materials and Methods

### 2.1. Experimental Facility and Data Collection

**Figure 1:**
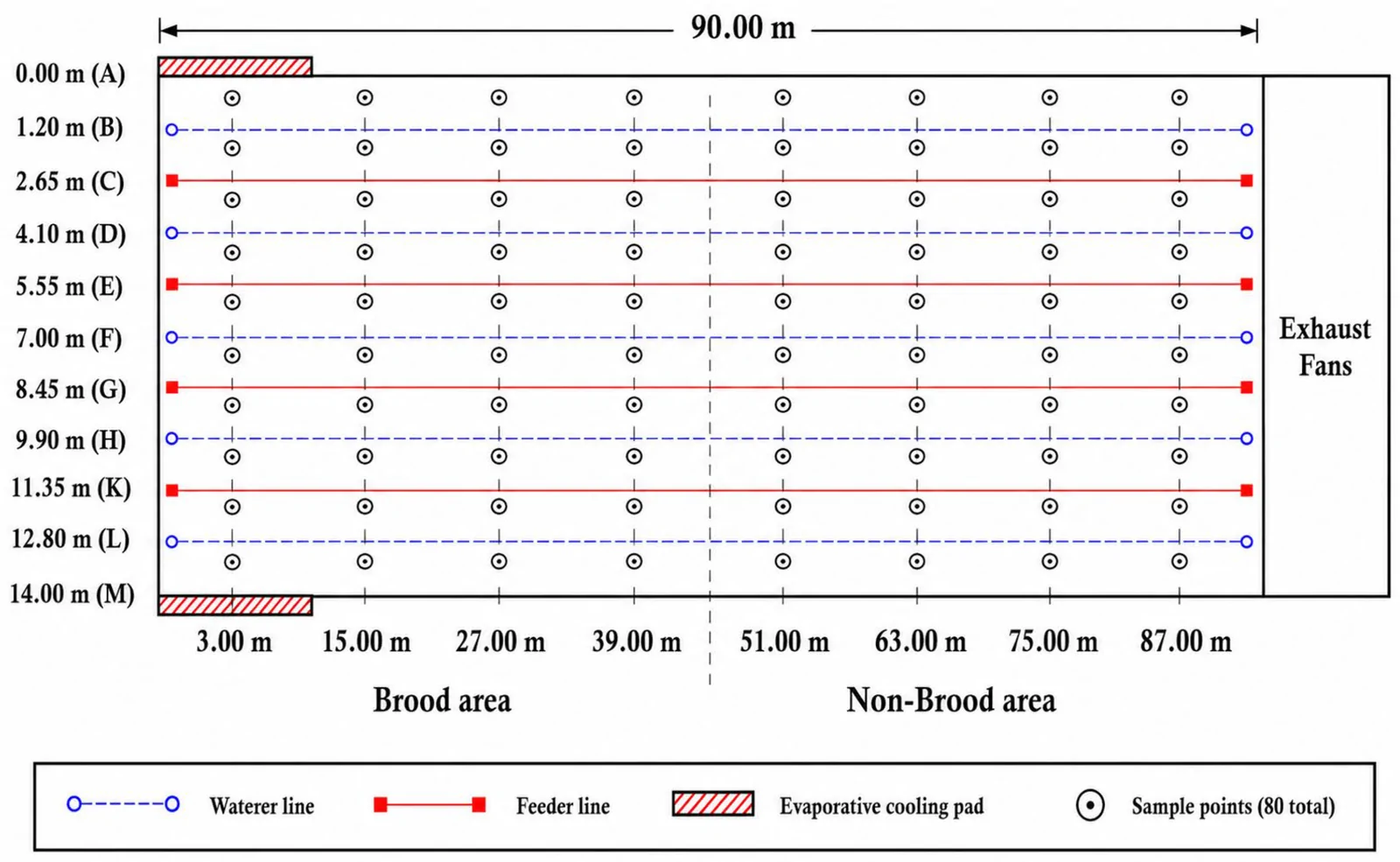
Schematic layout of the experimental broiler house and spatial sampling grid. The 40 locations corresponding to Y = 3, 15, 27, and 39 m were sampled on days 7, 21, and 42, whereas the additional locations at Y = 51, 63, 75, and 87 m were sampled only on days 21 and 42 after the birds gained access to the entire house.

This study was conducted in a commercial tunnel-ventilated broiler house located in Samsun, Türkiye (41°07′ N, 36°05′ E). The broiler house measured 90.0 m in length, 14.0 m in width, and 3.8 m in height. Environmental control, including feeding, drinking, lighting, heating, and mechanical ventilation, was automatically managed throughout each production cycle under commercial farming conditions. The ventilation system consisted of exhaust fans located at one end of the house and evaporative cooling pads installed at the opposite end (Figure 1).

Commercial Ross 308 broilers were reared from one day of age until market age (42 days). Before each production period, fresh rice-hull litter was uniformly applied to the floor at a depth of approximately 50 mm. During the brooding period (0–2 weeks), birds were confined to the brooding section of the house, after which they were allowed access to the entire building.

Data were collected from eight temporally distinct production periods (PP1–PP8) conducted sequentially in the same commercial broiler house under different seasonal conditions. Each production period involved a separate commercial flock, and fresh rice-hull litter was applied before the beginning of each cycle. Because all periods shared the same housing structure, ventilation system, geographic location, and general management environment, they were not considered externally independent populations. Measurements were performed on days 7, 21, and 42, representing the beginning, middle, and end of the production cycle, respectively. On day 7, birds were confined to the brooding area and measurements were obtained from 40 locations arranged in four longitudinal rows (Y = 3, 15, 27, and 39 m), with 10 transverse positions per row. On days 21 and 42, after birds gained access to the entire house, the same 40 locations were retained and four additional longitudinal rows (Y = 51, 63, 75, and 87 m) were added, resulting in 80 sampling locations. Spatial position was defined using Cartesian coordinates expressed in meters, with X representing position across the house width and Y representing position along the house length. Thus, 40 locations were directly comparable across all three sampling ages, and each production period contributed 200 observations in total (40, 80, and 80 observations on days 7, 21, and 42, respectively).

Air temperature (T), relative humidity (RH), and air velocity (V) were measured at two representative heights corresponding to bird level (0.40 m above the litter surface) and human level (1.80 m above the litter surface). At each sampling location, three consecutive measurements were obtained at each height, with each measurement representing the average value recorded over a 10 s interval. The V probe was aligned with the predominant airflow direction during each measurement to minimize directional bias. For each environmental variable, the three replicate measurements were first averaged separately at each height, and the resulting two height-specific means were subsequently averaged to obtain a single location-level T, RH, and V value used in the ML analyses. At each sampling location, T, RH, and V measurements were completed first, followed by thermal imaging of the litter surface; litter samples for LMC and LPH determination were collected immediately thereafter from the same local sampling area.

Following the environmental measurements, litter surface temperature (LT) was measured using a thermal imaging camera. The camera was positioned vertically above the litter surface at a fixed distance of approximately 0.40 m, and the emissivity was set to 0.95 throughout the measurements. Thermal images were acquired after ensuring that the measurement area was free of birds to minimize interference from body surface temperature. For each image, a rectangular region of interest (ROI) was manually defined near the center of the litter surface, corresponding to approximately 70% of the image width and 60% of the image height. Birds and other non-litter objects were excluded from the ROI, and the mean temperature within the selected ROI was recorded as the LT. The same ROI-selection criterion and image-analysis procedure were applied consistently across all sampling locations. Three consecutive thermal images were evaluated at each sampling location, and the mean of the three ROI-based temperature values was used as the location-level LT value in subsequent analyses.

Approximately 30 g of litter was collected from the upper 2–3 cm of the litter layer for each replicate. At each predefined sampling location, three field replicates were collected separately from adjacent points within the same local sampling area to account for small-scale litter heterogeneity. The three samples were kept separate during collection and laboratory processing rather than being pooled into a single composite sample. Immediately after collection, the samples were placed in sealed polyethylene bags and transported to the laboratory. Each replicate was homogenized individually before subsampling for LMC and LPH determination.

LMC was determined gravimetrically on a wet-weight basis. The initial wet mass of each subsample was recorded using an analytical balance, after which the samples were dried in an oven at 65 °C for 48 h and reweighed [20]. LMC was calculated from the mass loss during drying and expressed as a percentage of the initial wet mass.

LPH was determined using a 1:10 (*w*/*v*) litter-to-distilled-water suspension. The suspension was thoroughly mixed for 5 min and subsequently allowed to settle for 10 min before measurement. The pH meter was calibrated using standard buffer solutions at pH 4.0 and 7.0 before each measurement session, and pH measurements were performed at laboratory room temperature (approximately 20–25 °C) using a calibrated digital pH meter. The 1:10 extraction ratio and the mixing–settling procedure were consistent with previously reported poultry-litter pH measurement procedures [21]. For both LMC and LPH, each of the three field replicates was analyzed separately, and their values were averaged to obtain the location-level response used in the subsequent analyses.

For each observation, the corresponding bird number (BN), defined as the number of birds present in the broiler house on the sampling day after accounting for mortality and birds removed for early marketing, together with the sampling day (Age), production period (PP), and spatial coordinates (X and Y), was recorded. These environmental, temporal, seasonal, flock-related, and spatial variables constituted the predictor variables used for ML model development, whereas LMC and LPH served as the response variables.

All measuring instruments were calibrated according to the manufacturers’ recommendations before each measurement campaign. The specifications of the measuring instruments are summarized in Table 1.

### 2.2. Data Preprocessing and Predictor Scenarios

Prior to model development, a comprehensive quality assurance and quality control (QA/QC) procedure was conducted to ensure the reliability and consistency of the experimental dataset. The collected data were systematically examined for duplicate records, inconsistent entries, physically unrealistic measurements, and data formatting errors. All numerical variables were converted to a consistent numeric format and verified against their expected measurement ranges to identify potential data entry or sensor recording errors. No missing values were identified in either the predictor or response variables; therefore, no data imputation procedures were required.

Data collected on days 7, 21, and 42 were merged into a single analytical dataset to represent variation across the three sampled stages of the production cycle. Sampling day was encoded as the Age variable, and each observation was linked to its corresponding production period (PP1–PP8). Following data integration, a unique identification number was assigned to each observation to maintain traceability throughout preprocessing, cross-validation, and prediction analyses.

Because the spatial sampling domain differed between day 7 and the later sampling ages, Age and spatial coverage were partially confounded in the complete dataset. To evaluate the potential influence of this difference, an additional comparable-location sensitivity analysis was conducted using only the 40 sampling locations represented at all three ages. This restricted dataset comprised 960 observations (40 locations × 3 sampling ages × 8 production periods), thereby holding the spatial sampling domain constant across days 7, 21, and 42. Age-specific distributions of the environmental variables and litter responses in this subset were compared with those obtained from the complete dataset to determine whether the principal temporal patterns were preserved when spatial coverage was held constant.

Seasonal information was incorporated using three binary dummy variables representing Winter, Transition, and Summer. Each observation contained a single active seasonal indicator, thereby preserving the nominal nature of the seasonal categories without imposing an ordinal structure.

To investigate the contribution of different predictor groups, six progressively enriched predictor scenarios were defined (Table 2). The baseline scenario (S1) included T, RH, Age, and seasonal indicators. V, LT, BN, X-Y and the complementary litter property were then sequentially added to construct scenarios S2–S6. This scenario structure was designed to evaluate the incremental contribution of environmental, litter-related, flock-related, spatial, and complementary litter information. S6 included the complementary litter property (LPH for LMC prediction and LMC for LPH prediction) and was therefore defined as a litter-assisted theoretical performance-verification scenario rather than a practical standalone sensor-based deployment scenario.

Feature scaling was applied only to algorithms sensitive to differences in predictor magnitude. Predictor variables were standardized using Z-score normalization for SVR and MLP, whereas RF and XGB were trained using the original predictor scales. All preprocessing operations requiring parameter estimation, including standardization, were fitted exclusively within the corresponding training data and subsequently applied to the associated validation or test data.

**Figure 2:**
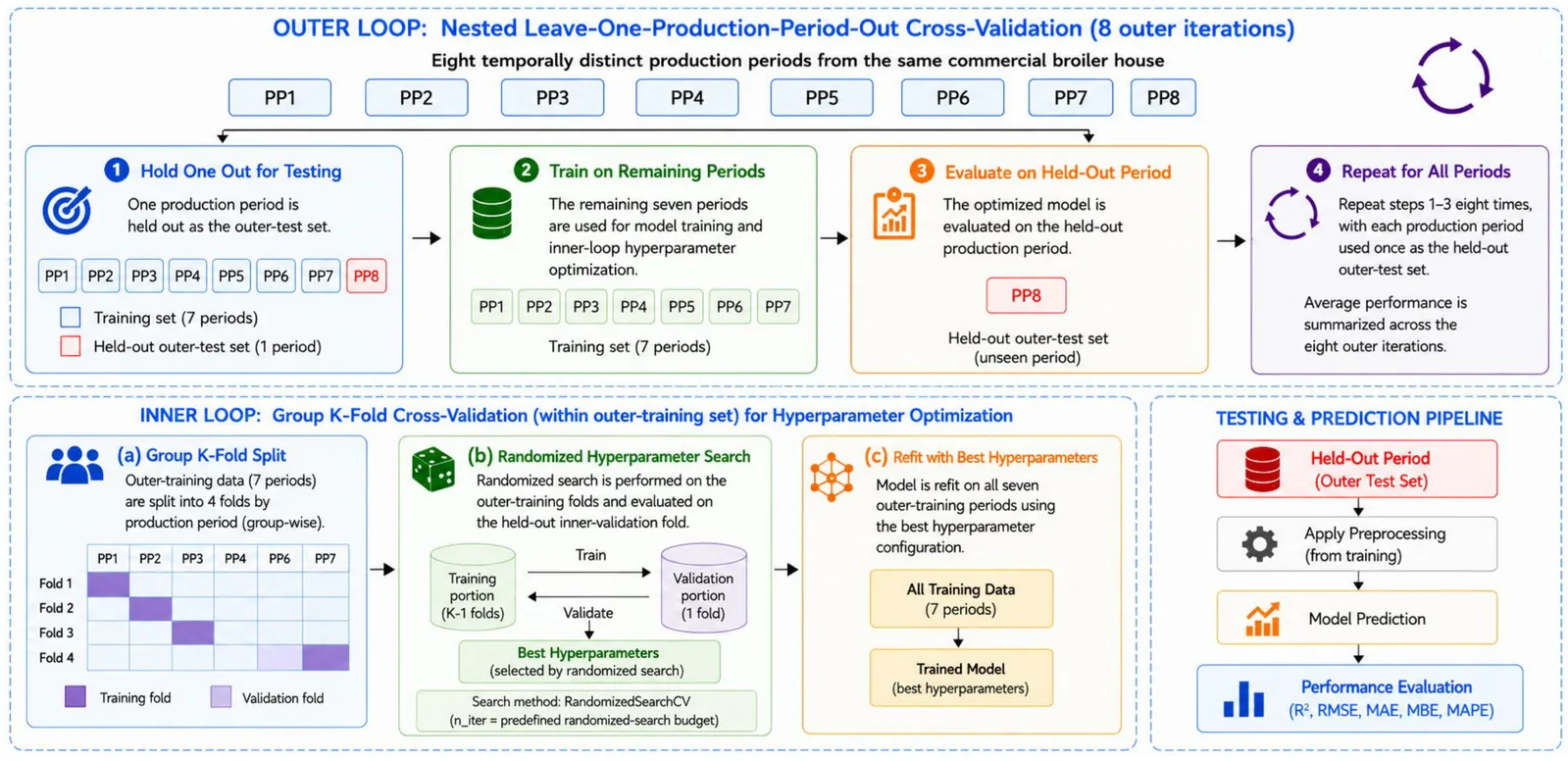
Workflow of the nested LOPO-CV framework used for model development, hyperparameter optimization, and grouped internal evaluation across held-out production periods.

The dataset had a grouped structure, with spatial observations nested within sampling days and production periods. Accordingly, PP was used as the grouping unit throughout model development and validation. Predictive performance was evaluated using a nested LOPO-CV framework, in which each PP served once as the held-out outer-test group while the remaining seven PPs formed the outer-training set. This design prevented observations from the same production cycle from being simultaneously represented in outer-training and outer-test data. Because all eight production periods originated from the same commercial broiler house, the procedure was interpreted as grouped internal validation rather than external validation (Figure 2).

### 2.3. Machine Learning Models

Four algorithms representing different ML paradigms were evaluated to predict LMC and LPH: SVR, RF, XGB, and MLP. These algorithms were selected to represent kernel-based learning, ensemble tree methods, gradient boosting, and artificial neural networks, respectively, thereby allowing comparative benchmarking under identical production-period-based validation conditions. The objective was to compare representative nonlinear regression approaches rather than to provide an exhaustive evaluation of all available ML algorithms. More complex deep-learning and sequential architectures were not included because the dataset consisted of a moderate number of tabular observations collected at three discrete sampling ages across eight production periods rather than continuous or high-frequency sequential measurements. Such models would require substantially larger and temporally denser datasets for a meaningful evaluation and were therefore considered beyond the scope of the present study.

SVR is a kernel-based learning algorithm that estimates nonlinear relationships by mapping the predictor variables into a higher-dimensional feature space through kernel functions while minimizing structural risk. In this study, the radial basis function (RBF) kernel was adopted because of its ability to model complex nonlinear relationships among environmental, flock-related, spatial, and litter-related predictors.

RF is an ensemble learning algorithm that constructs multiple decision trees using bootstrap sampling and random feature selection. The final prediction is obtained by averaging the outputs of the individual trees, thereby reducing prediction variance and improving predictive stability. Its ability to capture nonlinear relationships and interactions has supported its widespread application in agricultural prediction problems.

XGB is an optimized gradient boosting algorithm that sequentially constructs decision trees by minimizing a regularized objective function. The algorithm incorporates shrinkage, column subsampling, and regularization to control model complexity and reduce overfitting. Owing to its computational efficiency and ability to capture complex nonlinear relationships, XGB has been widely applied to regression problems.

The MLP is a feed-forward artificial neural network composed of interconnected neurons arranged in input, hidden, and output layers. During training, network weights are iteratively updated using backpropagation to minimize prediction error. Owing to its nonlinear activation functions, MLP is capable of approximating highly complex relationships between predictor variables and target responses.

All algorithms were implemented within a common computational framework and fitted separately for each target variable and predictor scenario. The same production-period-based partitions were used for all algorithms within a given analysis, ensuring that comparative benchmarking was based on identical outer-training and held-out outer-test groups.

### 2.4. Hyperparameter Optimization

Hyperparameter optimization was conducted exclusively within the outer-training set of each nested LOPO-CV iteration using RandomizedSearchCV. For each target variable, predictor scenario, and ML algorithm, candidate hyperparameter configurations were evaluated using four-fold Group K-Fold CV, with PP specified as the grouping variable. Thus, all observations belonging to the same PP remained together within each inner fold.

For each sampled configuration, RMSE was evaluated across the four inner-validation folds, and the configuration with the lowest mean inner-validation RMSE was selected. The selected configuration was then refitted using the complete outer-training dataset before predictions were generated for the corresponding held-out PP.

Algorithm-specific search spaces were defined to cover relevant levels of model complexity, regularization, learning rate, kernel or tree structure, and sampling behavior, as applicable to each algorithm. The randomized-search budgets were 10 configurations for SVR, 12 for RF, 40 for XGB, and 12 for MLP. These algorithm-specific budgets reflected differences in the size and complexity of the corresponding search spaces. A fixed random seed of 42 was used for randomized hyperparameter sampling and stochastic model components to improve computational reproducibility. The complete hyperparameter search spaces and fold-specific selected configurations are provided in Supplementary Spreadsheet S1, while model-specific convergence and stopping criteria are specified in the accompanying Supplementary Python Script.

### 2.5. Model Evaluation and Statistical Analysis

Model performance was quantified using the coefficient of determination (R^2^), root mean square error (RMSE), mean absolute error (MAE), mean bias error (MBE), and mean absolute percentage error (MAPE). These metrics were used to characterize the linear agreement between observed and predicted values, prediction-error magnitude, systematic bias, and relative prediction error. For MAPE, observations with an observed response equal to zero were excluded from the percentage-error calculation; however, no zero values occurred for either LMC or LPH in the present dataset. Mathematical expressions and definitions of the performance metrics are provided in Supplementary Table S1.

Outer-training performance was calculated after refitting the hyperparameter configuration selected in the inner loop using the complete outer-training dataset and therefore represents refit model performance rather than inner-validation performance. Held-out outer-test performance was calculated exclusively from predictions generated for the PP excluded in the corresponding outer LOPO iteration.

To characterize variation in predictive performance among production periods, each metric was retained separately for the eight held-out outer-test folds. For each target variable, predictor scenario, and ML algorithm, fold-specific values were summarized using the mean, standard deviation (SD), median, interquartile range (IQR), minimum, and maximum. A two-sided 95% confidence interval (CI) for the mean was calculated using Student’s *t* distribution. Thus, PP, rather than the individual spatial observation, was the unit used for summarizing uncertainty in outer-fold predictive performance.

Analytical repeatability of LMC and LPH measurements was evaluated using the three replicate measurements obtained at each sampling location. Within-location SDs were calculated for each response variable, followed by pooled within-location repeatability SDs and corresponding coefficients of variation (CV). Because the response values used for model development represented the mean of three replicates, the repeatability-related standard uncertainty of the location-level mean was estimated as the pooled repeatability SD divided by the square root of three. Manufacturer-specified instrument accuracies were considered separately and were not interpreted as complete method-level measurement uncertainty, which may additionally include field sampling, sample preparation, homogenization, calibration, and other sources.

Algorithm and predictor-scenario comparisons based on outer-fold performance were interpreted as exploratory benchmarking rather than as a separate final-model-selection procedure. Because the same PPs served as outer-test groups for all algorithms within a given target and scenario, between-model differences were also examined on a paired fold basis. For each performance metric, pairwise algorithm differences were calculated within each held-out PP and summarized across the eight folds using the mean paired difference and its two-sided 95% CI. Model rankings based on R^2^, RMSE, MAE, MAPE, and the absolute value of MBE were retained only as descriptive summaries and were not interpreted as inferential evidence of model superiority.

Predictions from the eight held-out outer-test folds were additionally concatenated to form pooled out-of-fold (OOF) predictions. Each observation therefore contributed to the pooled OOF dataset only when its corresponding PP was excluded from model development. R^2^, RMSE, MAE, MBE, and MAPE were calculated from the pooled OOF predictions separately from the mean of the eight-fold-specific values. Fold-specific metrics, uncertainty summaries, paired algorithm differences, pooled OOF metrics, and complete observation-level OOF predictions are provided in Supplementary Spreadsheet S3.

Permutation feature importance was calculated separately within each outer LOPO fold using the corresponding held-out outer-test PP. For each fitted model, each input variable was randomly permuted 30 times while the remaining inputs were left unchanged. Importance was evaluated using negative RMSE scoring, such that larger positive values indicated greater deterioration in predictive performance following permutation. A fixed random seed of 42 was used for reproducibility. Fold-specific importance values were retained and summarized across the eight outer folds using the mean and SD. Negative importance values were retained because they may indicate negligible or unstable predictive contribution. Permutation importance was interpreted as a model- and dataset-specific measure of predictive contribution rather than evidence of causality. Results are provided in Supplementary Spreadsheet S2.

Because the eight PPs were conducted sequentially in the same broiler house, possible dependence between successive flocks was examined at the PP level. Chronologically ordered PP-level means of LMC, LPH, T, RH, V, and LT were evaluated using lag-1 Pearson and Spearman correlations, together with the Durbin–Watson statistic, as descriptive indicators of first-order serial dependence. Given the small number of production periods (n = 8), these analyses were considered exploratory and were not used to establish statistical independence.

Between-period heterogeneity was further characterized by summarizing LMC and LPH separately for each PP using the number of observations, mean, SD, median, IQR, minimum, and maximum. In addition, a comparable-location sensitivity analysis was performed using only the 40 sampling locations represented at all three sampling ages. Age-specific descriptive statistics for T, RH, V, LT, LMC, and LPH in this common-location subset were compared descriptively with those from the complete dataset to assess whether the principal age-related patterns were preserved when the spatial sampling domain was held constant.

Pearson correlation analysis was used to describe linear relationships among continuous variables. These correlations were used only for descriptive characterization and not for predictor selection because the predictor scenarios were predefined according to the study design.

All computational analyses were performed using Python 3.11.7 (Anaconda distribution) on a 64-bit Windows 11 operating system. The computational workflow included NumPy 2.4.6, pandas 3.0.5, SciPy 1.17.1, scikit-learn 1.9.0, XGBoost 2.1.3, statsmodels 0.14.6, Matplotlib 3.11.0, and openpyxl 3.1.5 for data preprocessing, model development, statistical analysis, visualization, and data reporting. A fixed random seed of 42 was used for stochastic procedures to improve computational reproducibility. Detailed hyperparameter search spaces, search budgets, model-specific stopping criteria, and additional implementation details are provided in the Supplementary Analysis Script accompanying this manuscript.

## 3. Results

### 3.1. Descriptive Statistics of the Experimental Dataset

The combined experimental dataset comprised 1600 observations collected from eight temporally distinct commercial broiler production periods (PP1–PP8). Measurements recorded on days 7, 21, and 42 were merged into a single analytical dataset prior to model development. The dates of each production period, flock sizes, and additional production-cycle information are presented in the Supplementary Material (Table S2). Following data integration and production-period assignment, all observations met the predefined quality-control criteria and were included in the subsequent analyses.

Each PP contributed 200 observations. The complete dataset therefore comprised 320 observations on day 7 and 640 observations on each of days 21 and 42, reflecting the expansion of the sampling domain after the brooding phase.

Table 3 summarizes the descriptive statistics of the predictor and response variables according to sampling age. Clear changes in the environmental conditions were observed across the production cycle. Mean T decreased from 29.54 °C on day 7 to 21.74 °C on day 42, whereas mean RH increased from 58.70% to 68.80%. Mean V increased from 0.17 m s^−1^ on day 7 to 0.81 m s^−1^ on day 42 and showed greater variability at the later sampling ages. In contrast, T showed comparatively lower within-age variability.

Litter properties also differed across sampling ages. Mean LMC increased from 21.33% on day 7 to 31.39% on day 21 and 34.41% on day 42. Similarly, mean LPH increased from 6.52 to 7.64 and 7.81 across the same sampling ages. Mean LT varied comparatively less, ranging from 27.08 to 28.71 °C.

To assess whether these age-related patterns were influenced by the difference in spatial coverage among sampling days, the analysis was repeated using only the 40 locations represented at all three ages. The comparable-location subset contained 960 observations, with 320 observations at each sampling age. Mean LMC values in this subset were 21.33%, 31.04%, and 34.25% on days 7, 21, and 42, respectively, compared with 21.33%, 31.39%, and 34.41% in the complete dataset. Corresponding mean LPH values were 6.52, 7.64, and 7.80 in the comparable-location subset and 6.52, 7.64, and 7.81 in the complete dataset. Thus, restricting the descriptive analysis to a constant spatial sampling domain resulted in only minor changes in the observed response distributions, while the principal age-related patterns were preserved. Complete descriptive statistics for the comparable-location analysis are provided in Supplementary Table S3.

The replicate measurements showed low within-location analytical variability for both response variables. The pooled within-location repeatability SD was 0.146 percentage points for LMC and 0.0287 pH units for LPH, corresponding to pooled CV values of 0.476% and 0.383%, respectively. Because the modeled responses were based on the mean of three replicates, the corresponding repeatability-related standard uncertainties of the location-level means were 0.084 percentage points for LMC and 0.0166 pH units for LPH. These repeatability estimates were substantially smaller than the corresponding outer-fold prediction errors. Detailed age-specific repeatability results are provided in Supplementary Table S4.

Between-period distributions of LMC and LPH were also examined separately for PP1–PP8 and are provided in Supplementary Table S5. Exploratory analysis of successive production periods did not reveal a clear or consistent first-order serial pattern. Lag-1 Pearson correlations were −0.299 for LMC and −0.065 for LPH, while the corresponding Spearman correlations were −0.250 and 0.000. The Durbin–Watson statistics were 2.38 for LMC and 1.83 for LPH. No consistent positive lag-1 pattern was evident for T, RH, V, or LT. Given the limited number of production periods (n = 8), these results were interpreted descriptively and were not considered evidence of statistical independence. Complete serial-dependence results are provided in Supplementary Table S6.

Overall, the descriptive analyses indicated substantial variation in environmental and litter conditions across sampling ages and production periods. The comparable-location sensitivity analysis indicated that the principal age-related patterns in LMC and LPH were preserved when the descriptive comparison was restricted to locations represented at all three sampling ages.

### 3.2. Correlation Analysis

**Figure 3:**
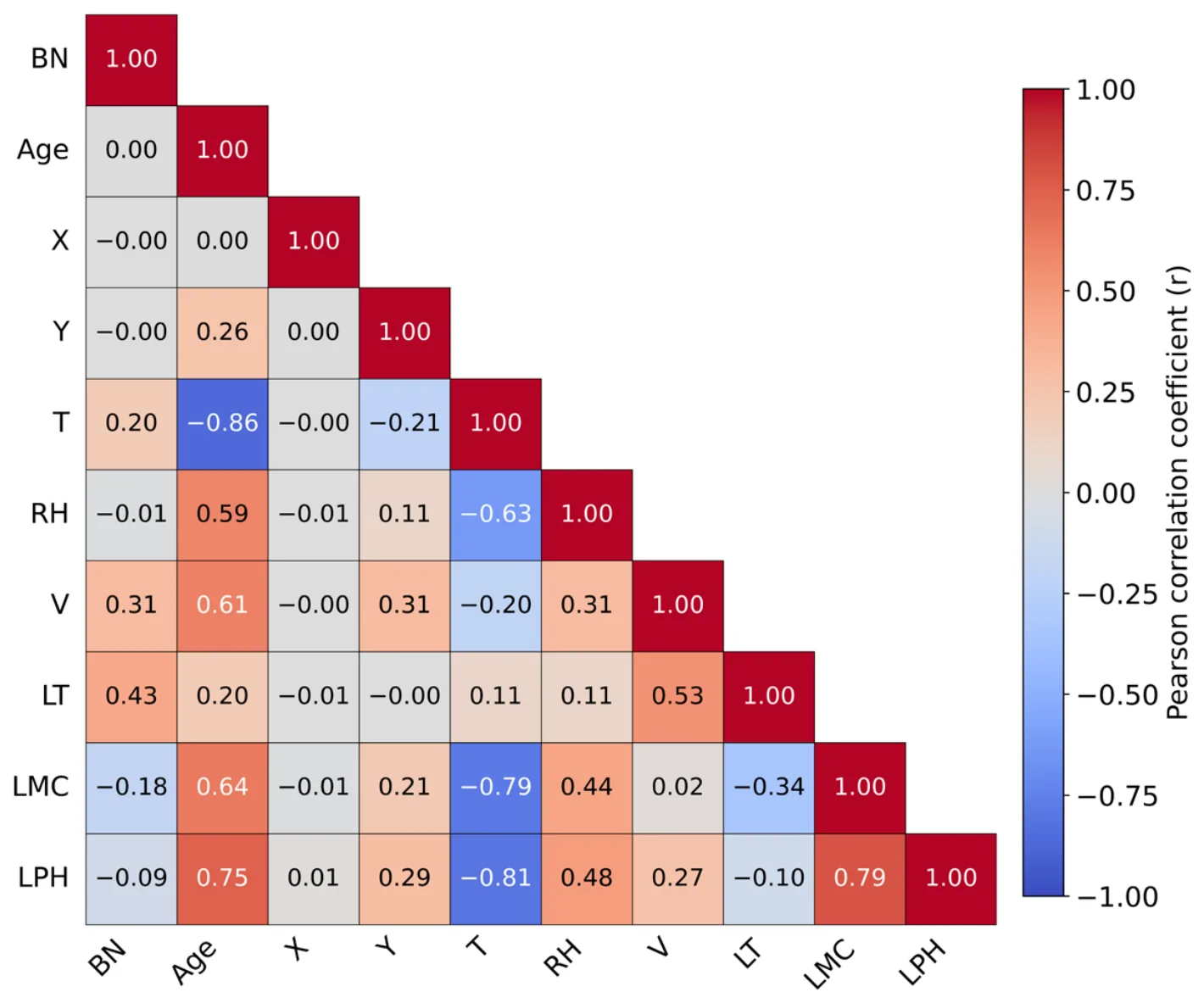
Pearson correlation matrix of the continuous predictor variables and the two response variables used for ML model development. T, air temperature; RH, relative humidity; V, air velocity; LT, litter temperature; BN, bird number; LMC, litter moisture content; LPH, litter pH; X and Y, spatial coordinates within the broiler house.

Figure 3 presents the Pearson correlation matrix among the continuous predictor variables and the two response variables used for ML model development. The correlation coefficients ranged from negligible to strong, indicating substantial variability in the relationships among the environmental, temporal, spatial, and litter-related variables.

Among the predictor variables, Age exhibited the strongest relationships with the environmental variables. Age was strongly negatively correlated with T (r = −0.86), whereas positive correlations were observed with V (r = 0.61) and RH (r = 0.59). T and RH also showed a moderately strong negative correlation (r = −0.63).

The two response variables were strongly positively correlated with each other (r = 0.79), indicating that increases in LMC were generally associated with increases in LPH. LMC exhibited a strong negative correlation with T (r = −0.79) and a moderate positive correlation with Age (r = 0.64) and RH (r = 0.44). In contrast, its relationships with V (r = 0.02), LT (r = −0.34), X (r = −0.01), and Y (r = 0.21) were weak to moderate.

Similarly, LPH was strongly negatively correlated with T (r = −0.81) and strongly positively correlated with Age (r = 0.75). Moderate positive correlations were observed between LPH and RH (r = 0.48), whereas weaker positive relationships were identified with V (r = 0.27) and Y (r = 0.29). The association between LPH and LT was weak (r = −0.10).

The spatial coordinates showed negligible linear relationships with most variables. The X-coordinate exhibited correlation coefficients close to zero for all variable pairs, while the Y-coordinate displayed only weak correlations, the highest being with V (r = 0.31), LPH (r = 0.29), and Age (r = 0.26). Likewise, BN showed generally weak to moderate correlations with the remaining variables, with the largest coefficients observed for LT (r = 0.43), V (r = 0.31), and T (r = 0.20).

The correlation analysis was used solely to describe linear associations among the measured variables and was not used for predictor selection, because the predictor scenarios had been predefined according to the experimental design. These associations were not interpreted as causal relationships. In particular, Age represented the sampling stage within the production cycle and covaried with other time-varying production conditions; moreover, spatial sampling coverage differed between day 7 and days 21 and 42. Therefore, correlations involving Age should be interpreted descriptively rather than as isolated effects of bird age.

### 3.3. LMC Prediction Performance

The predictive performances of the four ML algorithms for LMC under the six predictor scenarios are summarized in Table 4. The corresponding training performance results are provided in the Supplementary Material (Table S7). Model performance was evaluated using nested LOPO-CV across eight held-out production periods within the same commercial broiler house and is therefore presented as the mean ± SD across the eight outer-test folds. Detailed fold-specific outer-test values, across-period uncertainty summaries, paired between-algorithm differences, and pooled OOF performance results are provided in Supplementary Spreadsheet S3.

Using the baseline predictor set (S1), all four algorithms exhibited comparable predictive performance, with R^2^ values ranging from 0.737 to 0.752 and RMSE values between 3.282 and 3.361. RF achieved the highest R^2^ (0.752 ± 0.185), whereas SVR produced the lowest MAE (2.618 ± 0.797) and MAPE (8.945 ± 2.201%). The MBE values were close to zero for all algorithms, indicating negligible systematic prediction bias.

The inclusion of additional predictor variables generally improved model performance from S1 to S3, although the magnitude of improvement depended on the learning algorithm and evaluation metric. Incorporating V in S2 increased R^2^ for several models, particularly XGB, whose mean outer-fold R^2^ increased from 0.737 to 0.786. The subsequent addition of LT in S3 resulted in XGB achieving the highest mean outer-fold R^2^ among all evaluated configurations (0.796 ± 0.118), with RMSE = 3.082 ± 0.956, MAE = 2.503 ± 0.818, and MAPE = 8.552 ± 2.508%. In contrast, the performances of RF and SVR changed only marginally between S2 and S3.

The incorporation of BN (S4) did not result in consistent improvements across all algorithms. Within S4, XGB yielded the highest mean R^2^ (0.793 ± 0.088), whereas MLP exhibited a substantial deterioration in predictive performance, yielding the lowest R^2^ (0.695 ± 0.183) together with the highest RMSE (5.156 ± 2.792), MAE (3.995 ± 1.714), and MAPE (14.840 ± 8.272%). Moreover, MLP produced comparatively large SD values, indicating greater variability in predictive performance across the held-out production periods.

Adding spatial coordinates (X and Y) in S5 led to only modest changes in model performance. XGB under S5 yielded the lowest RMSE (3.023 ± 0.755) and MAPE (8.356 ± 2.433%) across all evaluated configurations, while its MAE (2.457 ± 0.638) was also relatively low. Its mean outer-fold R^2^ (0.781 ± 0.127) was slightly lower than that obtained under S3. The remaining algorithms showed only modest changes relative to S4.

When LPH was included as an additional predictor in S6, SVR showed the lowest MAE among all evaluated LMC configurations (2.433 ± 0.692), with R^2^ = 0.780 ± 0.124 and RMSE = 3.075 ± 0.872. However, comparison across all predictor scenarios revealed metric-specific trade-offs. XGB under S3 achieved the highest mean outer-fold R^2^ (0.796 ± 0.118), whereas XGB under S5 yielded the lowest RMSE (3.023 ± 0.755) and MAPE (8.356 ± 2.433%). Accordingly, no single algorithm–scenario combination was uniformly superior across all performance criteria.

The progressive enrichment of the predictor set improved LMC prediction primarily from S1 to S3, whereas the subsequent addition of BN and spatial coordinates resulted in algorithm- and metric-dependent changes rather than uniform improvement. S6 incorporated the complementary litter property (LPH) and was therefore interpreted as a litter-assisted theoretical performance-verification scenario rather than a practical standalone sensor-based deployment scenario. Overall, the outer-fold results indicate metric-specific trade-offs among algorithms and predictor scenarios rather than a uniquely optimal configuration.

To further illustrate the predictive characteristics of a representative high-performing model, additional visual analyses were performed, including descriptive model ranking, measured-versus-predicted scatter plots, and production-period-specific performance heat maps.

#### 3.3.1. Overall Model Ranking

**Figure 4:**
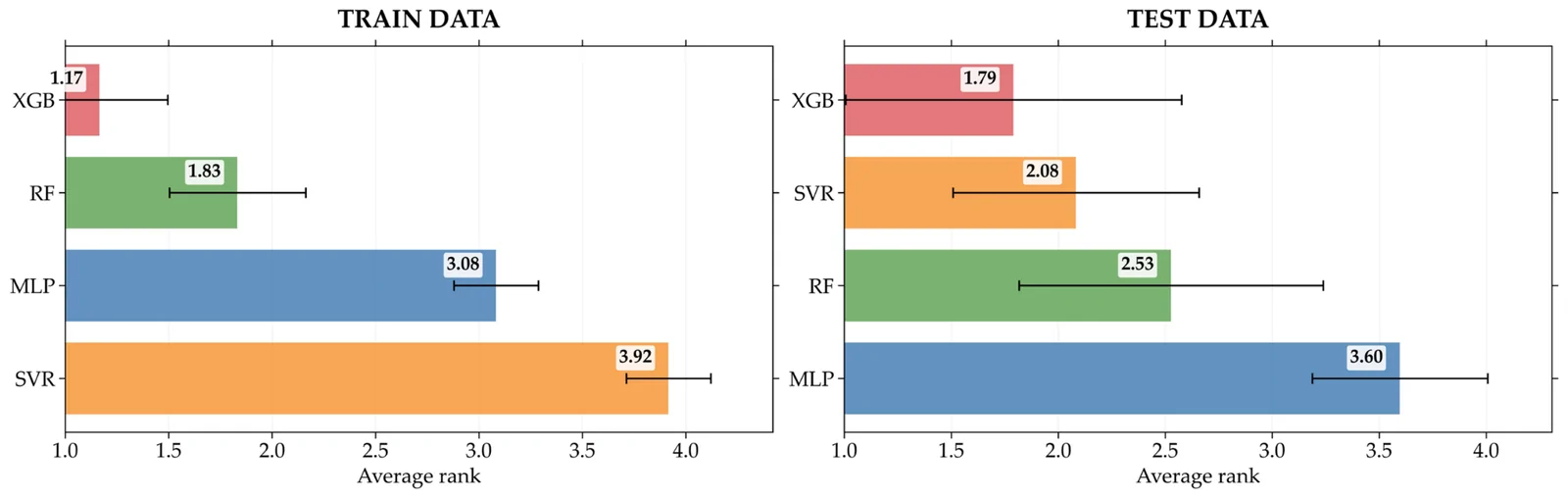
Descriptive mean ranking (±SD) of the four machine-learning algorithms for LMC prediction across the six predictor scenarios.

Figure 4 summarizes the overall ranking of the four ML algorithms based on five performance metrics (R^2^, RMSE, MAE, MAPE, and |MBE|) evaluated across the six predictor scenarios. For each scenario, the models were ranked separately for each performance metric. Rank 1 was assigned to the highest R^2^ value and to the lowest values of RMSE, MAE, MAPE, and |MBE|. The metric-specific ranks were then averaged to obtain an overall rank for each model within each scenario. Finally, the mean rank and its SD were calculated across all six scenarios. Lower mean rank values indicate a lower descriptive rank across the evaluated metrics, whereas the SD reflects variation in ranking across the six predictor scenarios.

For the training dataset, XGB achieved the lowest descriptive mean rank (1.17 ± 0.33), followed by RF (1.83 ± 0.33), MLP (3.08 ± 0.20), and SVR (3.92 ± 0.20). In the held-out outer-test results, XGB also had the lowest descriptive mean rank (1.79 ± 0.78), followed by SVR (2.08 ± 0.57), RF (2.53 ± 0.71), and MLP (3.60 ± 0.41). These rankings summarize relative performance across the evaluated metrics and scenarios and were not interpreted as inferential evidence of model superiority.

The descriptive ranking analysis showed that XGB generally achieved lower mean ranks across the evaluated LMC scenarios, whereas SVR and RF also demonstrated competitive performance depending on the predictor scenario and evaluation metric. MLP showed greater variability and generally higher mean ranks. These rankings were used only to summarize relative performance across the evaluated configurations and were not interpreted as inferential evidence of universal model superiority.

#### 3.3.2. Scatter Plot Analysis of the Representative XGB Model

**Figure 5:**
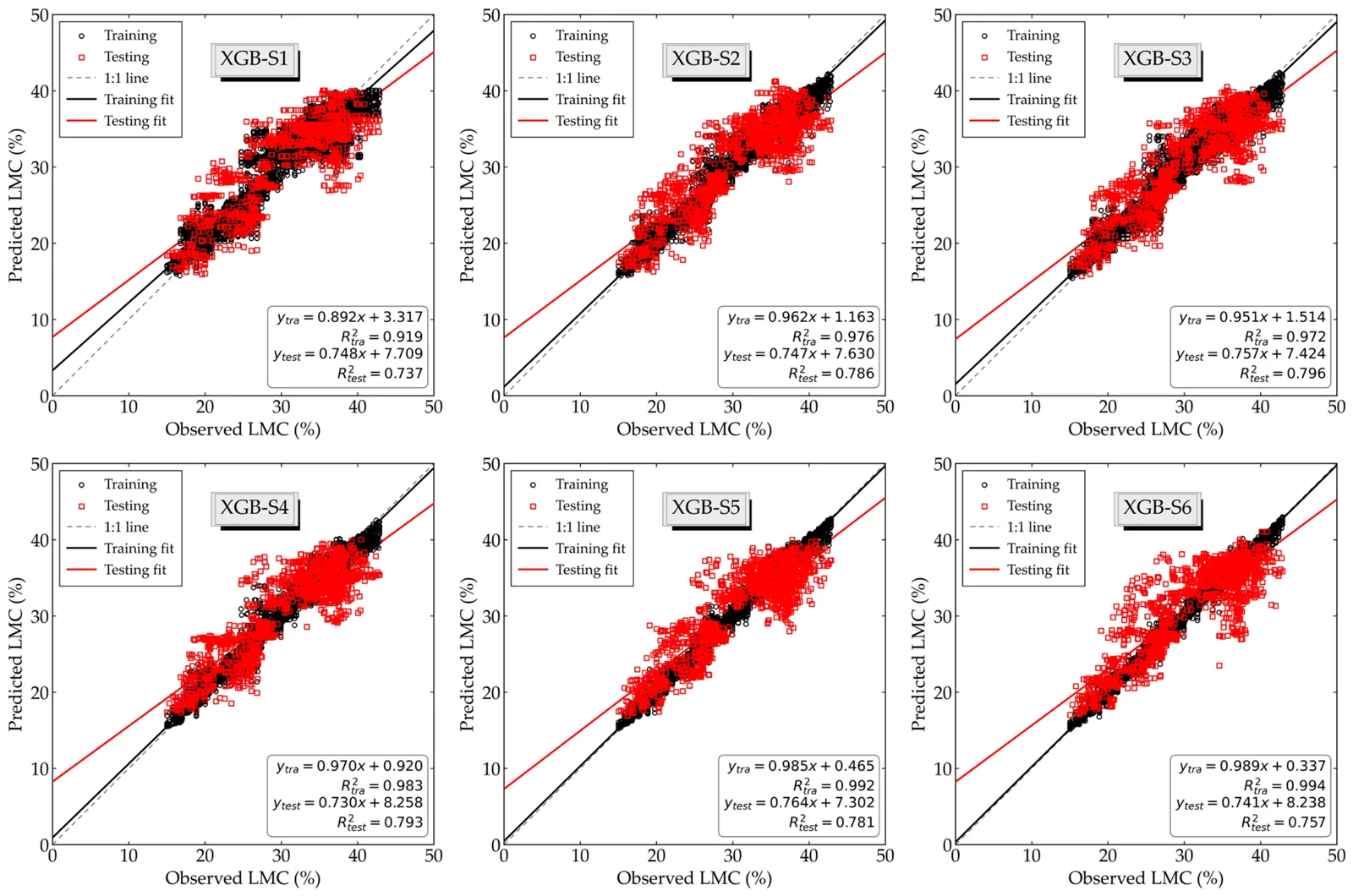
Measured versus predicted LMC values for the representative XGB model under the six predictor scenarios. Training and held-out outer-test results are identified explicitly in the corresponding panels.

Because XGB showed consistently competitive performance across the evaluated LMC scenarios and achieved the highest mean outer-fold R^2^ under S3, its measured-versus-predicted scatter plots are presented in the main manuscript (Figure 5). The corresponding plots for RF, SVR, and MLP are provided in the Supplementary Material (Figures S1–S3).

The predicted LMC values closely followed the 1:1 reference line for both the training and testing datasets across all six predictor scenarios, indicating good agreement with the measured values. Likewise, the fitted regression lines remained close to the reference line, confirming that the XGB model accurately captured the observed variation in LMC. Among the evaluated predictor scenarios, S3–S5 exhibited the most compact distributions around the 1:1 line, reflecting higher predictive accuracy and lower prediction errors than the remaining scenarios. For the training dataset, S5 achieved the highest coefficient of determination (R^2^ = 0.992), followed by S4 (R^2^ = 0.983). For the held-out outer-test dataset, S3 produced the highest agreement (R^2^ = 0.796), closely followed by S4 (R^2^ = 0.793), whereas S2 and S5 showed comparable predictive performance. In contrast, Scenarios S1 and S6 displayed slightly greater dispersion around the 1:1 reference line, particularly for observations with higher LMC values in the testing dataset. Nevertheless, the scatter plots revealed no evidence of systematic overestimation or underestimation across the evaluated scenarios, consistent with the near-zero MBE values reported in Table 4. These plots further illustrate the predictive behavior of the representative XGB model across the evaluated predictor combinations and held-out production periods within the studied house.

#### 3.3.3. Production-Period-Specific Performance Analysis

**Figure 6:**
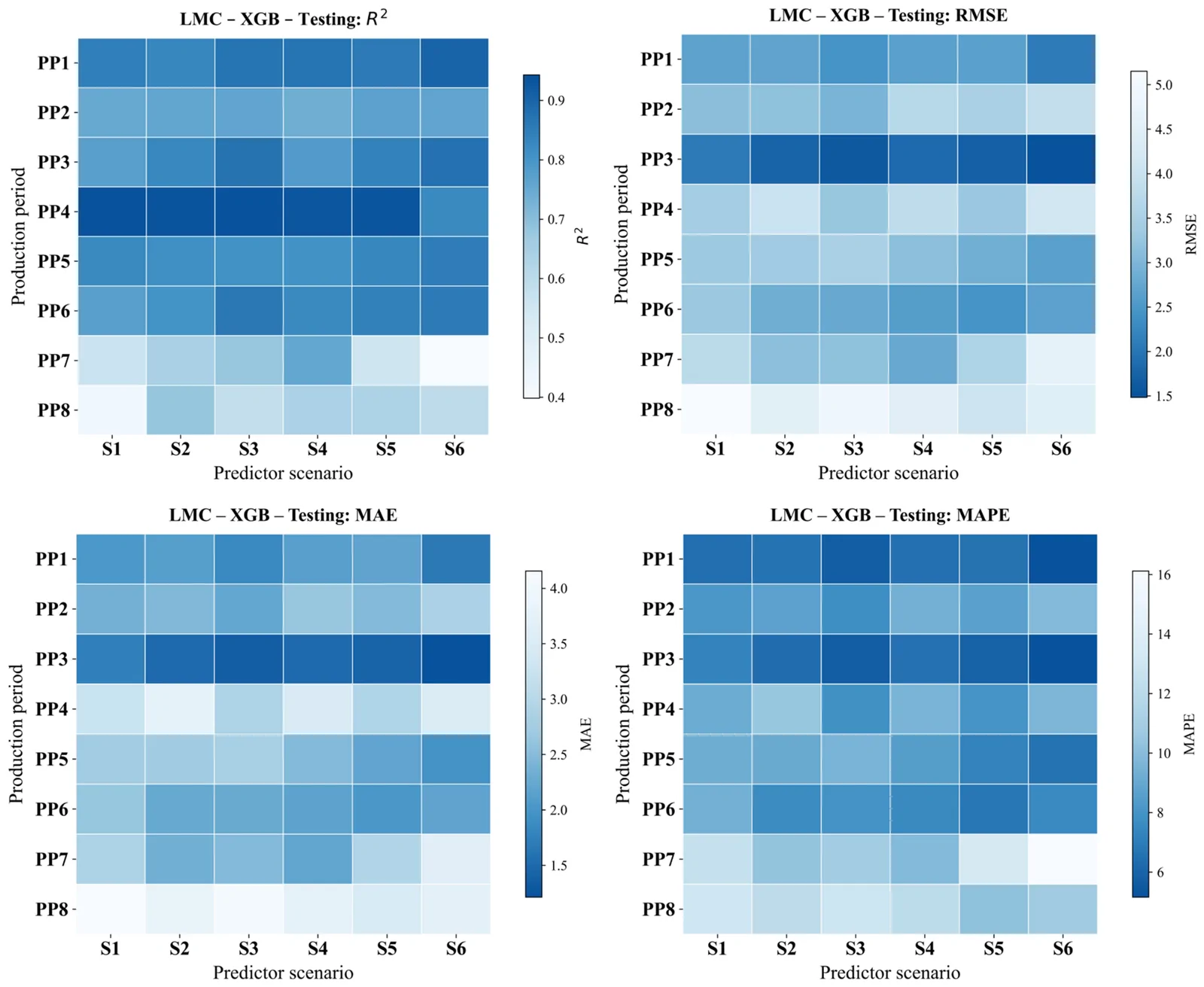
Production-period-specific held-out test performance of the XGB model for LMC prediction under the six predictor scenarios. Each PP represents one outer LOPO fold.

Production-period-specific heat maps for XGB are presented in Figure 6 to illustrate the variation in predictive performance across the held-out production periods. Corresponding results for RF, SVR, and MLP are provided in the Supplementary Material (Figures S4–S6).

PP3–PP6 exhibited relatively higher R^2^ values together with lower RMSE, MAE, and MAPE values than the remaining production periods, indicating higher predictive accuracy during these production cycles. In contrast, PP7 and PP8 generally showed lower R^2^ values and higher prediction errors across several predictor scenarios. Regarding production season, PP1, PP4, and PP5 were conducted during winter, PP2, PP6, and PP8 during the transition season, and PP3 and PP7 during summer. Production periods belonging to the same season did not consistently exhibit similar predictive performance. For example, PP3 achieved among the highest predictive accuracies, whereas PP7, despite also representing the summer season, showed comparatively lower performance. Likewise, the three winter production periods exhibited different levels of predictive accuracy across the evaluated predictor scenarios. The heat maps further indicate that prediction performance varied among production periods and predictor scenarios, suggesting that factors other than season also contributed to the observed differences in model performance.

### 3.4. LPH Prediction Performance

Table 5 summarizes the predictive performance of the four ML algorithms for LPH across the six predictor scenarios. The corresponding training performance results are provided in the Supplementary Material (Table S8). Overall, the evaluated algorithms produced relatively high R^2^ values and low prediction errors across most predictor scenarios. Corresponding fold-specific outer-test values, across-period uncertainty summaries, paired between-algorithm differences, and pooled OOF performance results are provided in Supplementary Spreadsheet S3.

Among the evaluated algorithms, RF showed consistently competitive performance for LPH across the six predictor scenarios. Its mean outer-fold R^2^ values ranged from 0.868 to 0.891, while RMSE ranged from 0.181 to 0.200. Under the litter-assisted S6 scenario, RF yielded R^2^ = 0.891 ± 0.110, RMSE = 0.181 ± 0.049, MAE = 0.139 ± 0.040, and MAPE = 1.922 ± 0.561%. MBE remained close to zero across the scenarios, indicating little systematic prediction bias. These results describe the relative performance observed across the outer folds and were not used to designate an independently validated final model.

XGB also showed consistently competitive predictive performance, with mean outer-fold R^2^ values ranging from 0.857 to 0.885. Within XGB, the highest R^2^ was observed under S6 (0.885 ± 0.115). Prediction errors were generally similar in magnitude to those obtained with RF across several scenarios, although their relative differences varied according to the evaluation metric and predictor composition.

SVR performed competitively when only a limited number of predictors were included. The highest R^2^ value for SVR was obtained in S1 (0.869 ± 0.132), after which predictive performance gradually declined as additional predictor variables were incorporated. The largest reductions were observed in S4–S6, where R^2^ decreased to 0.792, 0.773, and 0.788, respectively, accompanied by higher RMSE and MAE values.

MLP showed greater variability than the remaining algorithms. Although it achieved relatively high prediction accuracy in the simpler predictor scenarios, its performance fluctuated considerably across the six scenarios, particularly in S3–S5, where larger standard deviations and lower R^2^ values were observed. Prediction errors also increased compared with RF and XGB in these scenarios.

From the perspective of predictor composition, S1 already provided relatively high LPH predictive performance for all four algorithms. The addition of V in S2 produced only modest changes, whereas the inclusion of LT and the subsequent expansion of the predictor set resulted in more pronounced algorithm-dependent differences. Under S6, RF and XGB showed strong performance within the evaluated benchmark; however, the magnitude and direction of the differences among scenarios depended on both the algorithm and the performance metric. Accordingly, the outer-fold comparisons were interpreted as exploratory, metric-specific benchmarking rather than as evidence of a uniquely optimal predictor scenario.

To further summarize the relative behavior of the evaluated algorithms, descriptive model rankings, measured-versus-predicted plots, and production-period-specific performance heat maps were examined.

#### 3.4.1. Overall Model Ranking

**Figure 7:**
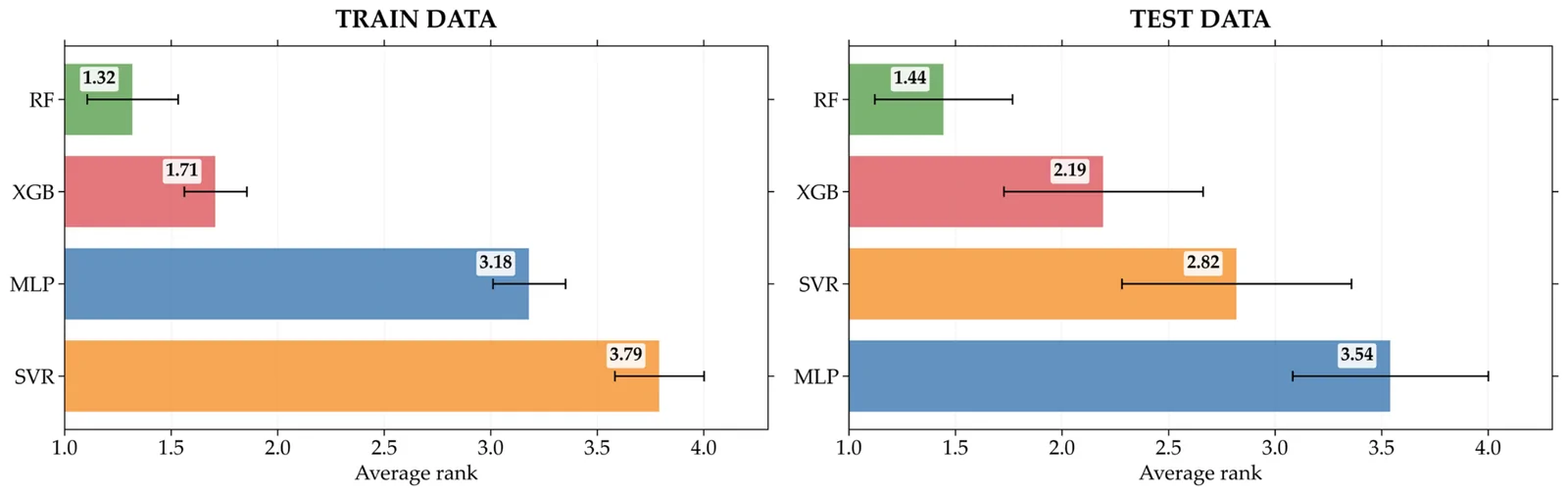
Descriptive mean ranking (±SD) of the four machine-learning algorithms for LPH prediction across the six predictor scenarios.

Figure 7 presents the overall ranking of the four ML algorithms for LPH prediction based on their predictive performance throughout the study. The ranking patterns were generally consistent between the training and testing datasets. In the training dataset, RF achieved the lowest average rank (1.32 ± 0.21), followed by XGB (1.71 ± 0.15), while MLP (3.18 ± 0.17) and SVR (3.79 ± 0.21) ranked third and fourth, respectively. A similar ordering was observed for the testing dataset, where RF again achieved the lowest average rank (1.44 ± 0.32), followed by XGB (2.19 ± 0.47), SVR (2.82 ± 0.54), and MLP (3.54 ± 0.46).

Compared with the training dataset, the relative differences among the algorithms became slightly smaller in the testing dataset, particularly between XGB and SVR. Nevertheless, RF consistently maintained the lowest average rank in both datasets. The relatively short error bars for RF indicate that its ranking varied only slightly across the six predictor scenarios, whereas the larger error bars for SVR and MLP reflect greater variability in their rankings.

The descriptive ranking analysis showed that RF generally achieved the lowest mean rank across the evaluated LPH scenarios, with XGB also maintaining competitive rankings. These rankings were used to summarize relative performance across the evaluated configurations and were not interpreted as inferential evidence of model superiority.

#### 3.4.2. Scatter Plot Analysis of the Representative RF Model

**Figure 8:**
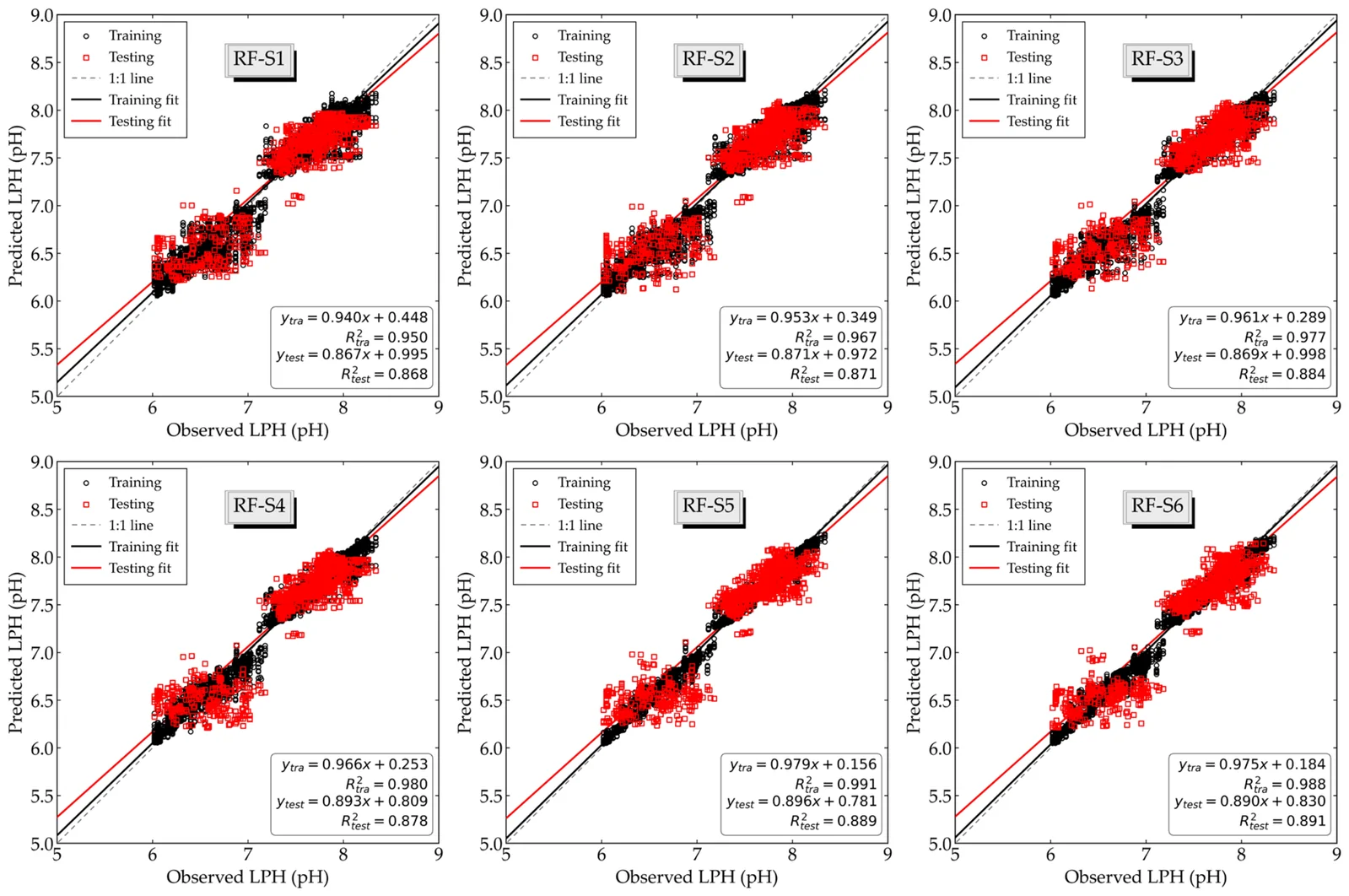
Measured versus predicted LPH values for the representative RF model under the six predictor scenarios. Training and held-out outer-test results are identified explicitly in the corresponding panels.

RF was selected for visualization in the main manuscript because it showed consistently competitive LPH performance and the lowest descriptive mean rank across the evaluated scenarios. Its measured-versus-predicted plots are presented in Figure 8, whereas the corresponding plots for XGB, SVR, and MLP are provided in the Supplementary Material (Figures S7–S9).

The scatter plots indicate that the RF model accurately reproduced the observed LPH values across all predictor scenarios. In both the training and testing datasets, most data points were closely distributed around the 1:1 reference line, indicating good agreement between the measured and predicted values.

The distribution of observations was not uniform across the entire LPH range. Most samples were concentrated between pH 7.0 and 8.0, where only small deviations between measured and predicted values were observed. A wider spread of points occurred at lower LPH values, particularly in the testing dataset; however, these observations represented only a small proportion of the dataset.

For the RF model shown in Figure 8, S6 exhibited the closest overall agreement between measured and predicted values, with S5 showing a similarly compact distribution around the 1:1 line. Nevertheless, the differences among scenarios were interpreted descriptively rather than as evidence of a uniquely optimal predictor configuration. The scatter plots indicate that the RF model provided reliable LPH predictions across the full range of observations, while differences in predictive performance among the evaluated predictor scenarios remained relatively small.

#### 3.4.3. Production-Period-Specific Performance Analysis

**Figure 9:**
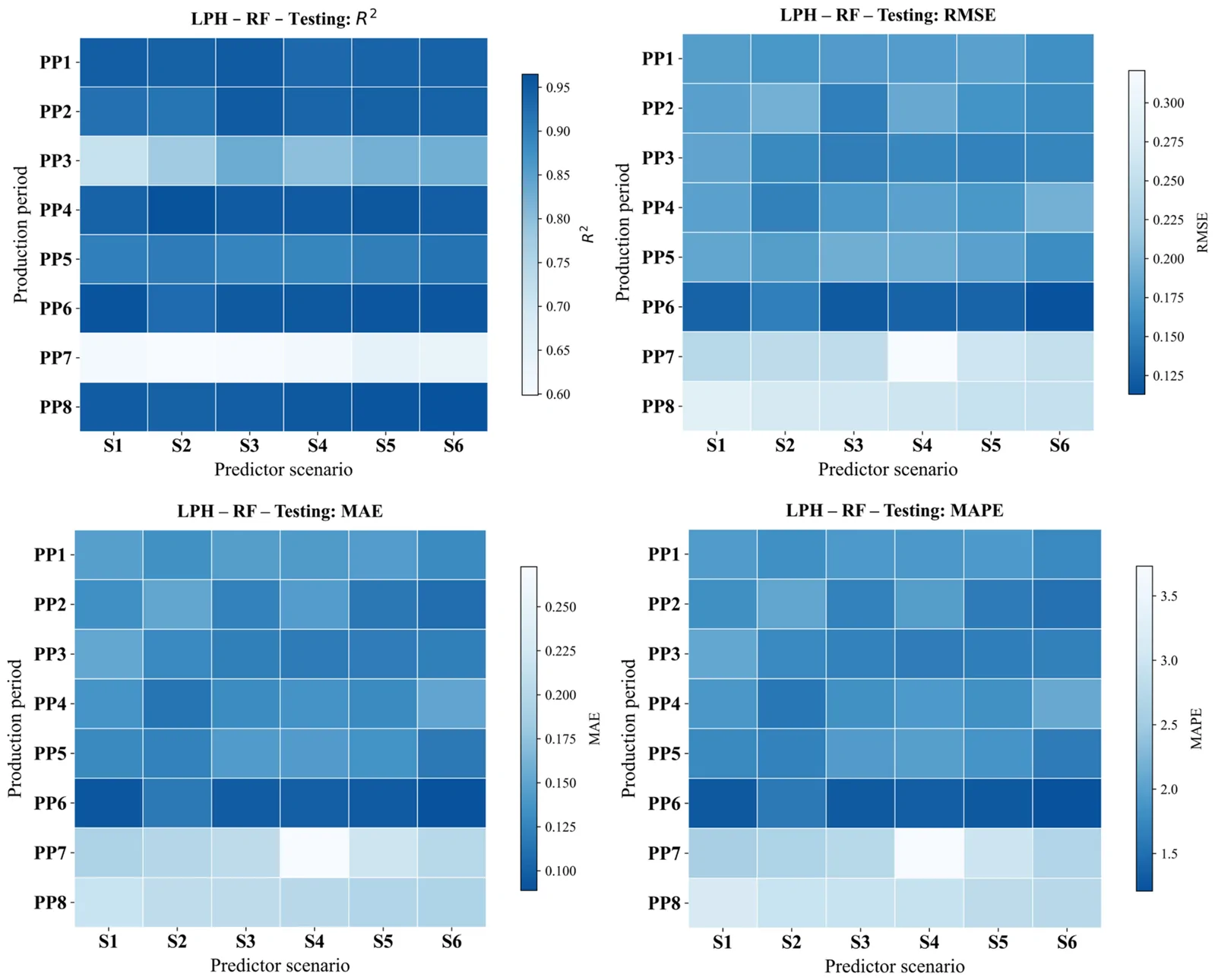
Production-period-specific held-out test performance of the RF model for LPH prediction under the six predictor scenarios. Each PP represents one outer LOPO fold.

Production-period-specific heat maps for the RF model are presented in Figure 9 to illustrate variations in LPH predictive performance across the held-out production periods. Corresponding heat maps for XGB, SVR, and MLP are provided in the Supplementary Material (Figures S10–S12).

Prediction accuracy remained relatively consistent for PP1–PP6, where most predictor scenarios produced high R^2^ values together with low RMSE, MAE, and MAPE values. Among these production periods, PP4 and PP6 consistently exhibited the most favorable predictive performance across the evaluated scenarios.

Greater variability was observed in the remaining production periods. PP7 consistently produced the lowest R^2^ values and the largest prediction errors regardless of the predictor scenario, whereas PP8 also showed reduced predictive performance compared with most of the other production periods. In contrast, the remaining production periods displayed only moderate differences among the six predictor scenarios.

Grouping the production periods according to season did not reveal a common pattern in prediction accuracy. Production periods belonging to the same season frequently exhibited different performance levels. For example, the two summer production periods (PP3 and PP7) showed markedly different prediction accuracies, while similar variability was observed among the winter and transition production periods.

The heat maps indicate that the predictive performance of the RF model remained relatively stable across most production periods, with the main exceptions being PP7 and, to a lesser extent, PP8. These findings suggest that variation in predictive performance was more closely associated with individual production periods than with a consistent seasonal pattern.

## 4. Discussion

### 4.1. Overall Predictive Performance

The present study showed that ML models could predict both LMC and LPH across held-out production periods within the studied commercial broiler house using environmental, flock-related, spatial, and litter-related information. These findings support the potential use of ML for data-driven litter monitoring in precision livestock farming, where complex interactions among environmental, biological, and management variables may limit the applicability of conventional statistical approaches. Previous studies have similarly reported that ML algorithms can capture nonlinear relationships in agricultural systems, although many relied on randomly partitioned datasets that may overestimate predictive performance because observations from the same production cycle can appear in both the training and testing datasets [11,22,23].

To address this limitation, the present study employed a nested LOPO-CV framework in which each production period served once as the held-out outer-test group. This strategy provided a more stringent within-house assessment by evaluating model performance on a complete production cycle excluded from preprocessing, hyperparameter optimization, and model fitting, thereby reducing the risk of information leakage between model development and outer-test evaluation. However, because all production periods originated from the same commercial broiler house, the held-out periods were not regarded as externally independent populations. Accordingly, the resulting performance estimates represent grouped internal validation across temporally distinct production periods within the studied house rather than external validation. Similar grouped validation approaches have recently been recommended for repeated or hierarchical agricultural datasets [24,25].

The comparative analysis indicated that algorithm performance depended on the response variable, predictor composition, and evaluation metric, supporting the general observation that model performance in agricultural prediction tasks is strongly data- and problem-dependent [26,27]. For LMC, XGB frequently showed competitive performance, particularly in terms of R^2^ and RMSE, whereas SVR achieved the lowest MAE under some predictor configurations. For LPH, RF generally showed competitive performance across several scenarios, with XGB also producing similar results under some predictor combinations. These patterns illustrate metric- and scenario-specific trade-offs among the evaluated algorithms rather than definitive superiority of a single model. Because algorithm and predictor-scenario comparisons were based on the same outer-fold evaluations used to characterize predictive performance, these findings were interpreted as exploratory benchmarking rather than as an independently validated final model selection.

SVR produced competitive predictions under several lower-dimensional predictor scenarios but showed more variable performance as additional predictors were introduced. MLP also showed greater variability across several scenarios, which may partly reflect the relatively limited number of production periods available for model development. Neural-network models generally benefit from larger and more diverse tabular datasets, whereas tree-based ensemble methods can remain competitive under more limited tabular-data settings [28,29].

These findings demonstrate that algorithm performance should be interpreted in relation to the prediction target, predictor composition, and evaluation metric rather than assuming that one ML algorithm is universally superior. The production-period-based validation framework provided a structured comparison of the evaluated algorithms across held-out production periods within the studied house, while broader generalization requires validation in additional commercial facilities.

### 4.2. Predictor Contributions and Mechanistic Interpretation

The predictor-scenario and permutation-importance analyses indicated that the predictive contribution of individual variables differed according to the target variable, algorithm, and predictor combination. Across the evaluated configurations, Age and T generally emerged as recurrently important predictors for both LMC and LPH, whereas the contribution of RH was smaller and more model-dependent. These patterns should be interpreted as predictive associations rather than causal effects, particularly because several predictors were correlated and Age also represented the sampling stage within the production cycle.

The predictive relevance of T and RH is consistent with the physical processes governing litter moisture dynamics. Litter drying depends on the transfer of water from the litter surface to the surrounding air. Higher T can increase evaporative potential, whereas higher RH reduces the capacity of the surrounding air to accept additional water vapor; the resulting moisture removal therefore depends on their interaction with ventilation and airflow [30]. This provides a plausible physical basis for the predictive contribution of T and, to a lesser extent, RH to LMC. However, their permutation-importance values should not be interpreted as direct estimates of their physical effects because correlated predictors can share predictive information.

Age should likewise be interpreted as a composite temporal predictor rather than as an isolated biological effect. As broilers grow, cumulative manure deposition, water intake and excretion, bird biomass, and ventilation demand change throughout the production cycle. These processes can progressively alter litter moisture and chemical conditions. Consistent with this temporal progression, mean LMC increased from 21.33% on day 7 to 31.39% on day 21 and 34.41% on day 42, while mean LPH increased from 6.52 to 7.64 and 7.81. Nevertheless, because the spatial sampling domain was smaller on day 7 than on days 21 and 42, Age remained partially confounded with sampling coverage and other time-varying production factors. The comparable-location sensitivity analysis showed that the main age-related response patterns were preserved when the same spatial locations were compared, but this does not establish a causal effect of bird age.

The relationships among LMC, T, and LPH also have a plausible biochemical basis. Adequate LMC facilitates microbial activity and nitrogen transformation, including degradation of nitrogen-containing excreta and formation of ammoniacal nitrogen. Litter pH is linked to these processes through acid–base equilibria, particularly the ammonium–ammonia system (NH_4_^+^ ⇌ NH_3_ + H^+^) [31]. Changes in pH alter the relative proportions of ammonium and ammonia, while T and LMC can further affect microbial transformations and ammonia volatilization. Thus, the predictive associations of T, RH, Age, LMC, and LPH are biologically plausible; however, the present observational study did not directly measure microbial nitrogen transformations or ammonia fluxes and therefore cannot establish these mechanisms experimentally.

The contributions of V, LT, BN, and spatial coordinates were generally more variable among algorithms and scenarios. Scenario-level changes in predictive performance after the addition of these variables should not be interpreted as direct measures of their individual importance because interactions and correlations among predictors may redistribute predictive information. In particular, the permutation-importance results indicated that some variables contributed little or inconsistently in individual model–scenario combinations. Spatial and flock-related information may nevertheless help represent local heterogeneity within the house, where airflow, bird distribution, manure deposition, and moisture accumulation vary spatially [32].

S6 incorporated the complementary litter property—LPH for LMC prediction and LMC for LPH prediction—and therefore provided a theoretical assessment of the additional predictive information available when one litter characteristic was already known. Its performance varied according to the target, algorithm, and evaluation metric and did not indicate a universally superior configuration. Accordingly, S6 should be regarded as a litter-assisted theoretical performance-verification scenario rather than a practical standalone sensor-based deployment scenario.

Finally, permutation importance should be interpreted cautiously when predictors are correlated because predictive information can be shared among variables, reducing or redistributing their individual importance values. The observed importance values therefore represent model- and dataset-specific measures of predictive contribution rather than evidence of causal effects. Complete permutation-importance results are provided in Supplementary Spreadsheet S2.

### 4.3. Practical Implications, Limitations and Future Research

The present findings provide a basis for the further development of data-driven litter-monitoring systems in commercial broiler production. From an engineering perspective, a prospective monitoring framework could combine routinely available T and RH measurements with V and litter-surface thermal information collected at representative locations within the house. These measurements could be transmitted to a local edge-computing unit or centralized monitoring platform, where the trained prediction model would generate updated estimates of litter condition for decision support. Predictor scenarios S1–S5 are more directly relevant to such sensor-oriented applications, whereas S6 requires prior knowledge of the complementary litter property and should therefore be regarded as a litter-assisted theoretical performance-verification scenario rather than a standalone deployment configuration. Sensor number, placement density, sampling frequency, calibration procedures, data-transmission requirements, and decision thresholds would need to be optimized prospectively before operational implementation.

Several limitations should be considered when interpreting the present results. All eight production periods were obtained from a single commercial broiler house with one broiler genotype and one litter material. Accordingly, the nested LOPO-CV framework demonstrates predictive robustness across held-out production periods within the studied house rather than external generalizability. Housing geometry, ventilation design, management practices, litter material, and regional climatic conditions can alter the relationships among T, RH, V, LMC, and LPH. Therefore, validation in houses with different structural characteristics, ventilation systems, litter types, management conditions, and climatic regions is required before broader application.

The temporal resolution of the present dataset also represents an important limitation. Measurements were obtained at three discrete sampling ages (days 7, 21, and 42), providing snapshots of litter conditions rather than a continuous representation of temporal dynamics. The descriptive results showed progressive increases in mean LMC and LPH across these sampling ages; however, the trajectory between sampling points could not be determined and may include nonlinear or short-term changes related to ventilation adjustments, drinking-water use, manure deposition, weather conditions, and management events. Thus, the current models should not be interpreted as dynamic time-series models.

Future studies should therefore collect temporally denser measurements at fixed spatial locations together with timestamped environmental, flock, weather, ventilation, and management information. Such datasets would enable the use of lagged predictors and dynamic modeling approaches, including autoregressive or state-space models as well as recurrent neural networks, temporal convolutional networks, and other sequence-learning architectures. For these applications, temporal validation should preserve chronological order—for example through blocked or rolling-origin evaluation—and, where possible, prospective testing in production periods and commercial houses not used during model development.

Additional limitations concern the observational design and measurement process. The spatial sampling domain differed between day 7 and days 21 and 42, so bird age and spatial coverage were partially confounded in the complete dataset. Although the comparable-location sensitivity analysis showed that the principal response patterns were preserved when the same spatial locations were considered, the design does not permit complete causal separation of bird age from other time-varying production and management factors. Likewise, the replicate analyses quantified analytical repeatability but did not represent complete method-level measurement uncertainty, which may also include variability arising from field sampling, sample preparation, homogenization, subsampling, and calibration.

Finally, the exploratory lag-1 correlation and Durbin–Watson analyses did not reveal a clear serial pattern across successive production periods, but the small number of periods (n = 8) limits the statistical power of these analyses and does not establish statistical independence. Algorithm and predictor-scenario comparisons were also based on the same outer-fold evaluations used to characterize predictive performance and should therefore be interpreted as exploratory benchmarking rather than definitive final-model selection. Independent prospective validation is required before selecting a model configuration for operational deployment or assessing potential effects on litter management, bird welfare, ammonia emissions, environmental control, or production sustainability.

## 5. Conclusions

This study developed and evaluated ML models for predicting LMC and LPH in a commercial mechanically ventilated broiler house using a nested LOPO-CV framework. Six predictor scenarios with progressively increasing complexity were investigated to evaluate the contributions of environmental, flock-related, spatial, and litter-related variables to prediction performance across held-out production periods within the studied commercial broiler house.

The results showed that predictive performance depended on the target variable, predictor composition, algorithm, and evaluation metric. For LMC, XGB frequently showed competitive performance, particularly in terms of R^2^ and RMSE, whereas SVR achieved the lowest MAE under some configurations. For LPH, RF generally showed competitive performance across several scenarios, with XGB producing similar results under some predictor combinations. These findings indicate metric- and scenario-specific trade-offs rather than definitive superiority of a single algorithm.

The inclusion of additional predictors did not produce uniform improvements across all algorithms and metrics. S6, which incorporated the complementary litter property, provided useful information for evaluating the theoretical predictive value of litter-assisted input but should not be interpreted as a practical standalone sensor-based deployment scenario.

The nested LOPO-CV framework reduced the risk of information leakage by evaluating each production period only after its complete exclusion from model development. However, because all production periods originated from the same commercial broiler house, the resulting performance estimates represent grouped internal validation across temporally distinct production periods within the studied house rather than external validation.

Overall, the findings support the feasibility of data-driven prediction of LMC and LPH under the conditions represented in this study. Further prospective validation is required across different commercial houses, litter materials, ventilation systems, management conditions, and climates before operational deployment. Potential effects on litter-management outcomes, bird welfare, ammonia emissions, environmental control, and production sustainability were not evaluated in the present study and should be investigated in future work.

## Figures and Tables

**Table 1:** Instruments and manufacturer-specified measurement ranges, accuracies, and resolutions used for environmental and litter measurements.

Variable	Instrument	Manufacturer	Measurement Range	Accuracy/Specification
T	Testo 605i thermo-hygrometer	Testo AG, Lenzkirch, Germany	−20–60 °C	±0.5 °C (0–60 °C); ±0.8 °C (−20–0 °C)
RH	Testo 605i thermo-hygrometer	Testo AG, Lenzkirch, Germany	0–100% RH	±2% RH (35–65% RH); ±3% RH (10–35 and 65–90% RH); ±5% RH (<10 or >90% RH), at 25 °C
V	PCE-423 hot-wire anemometer	PCE Instruments, Jupiter, FL, USA	0–30 m s^−1^	±5% of reading ±1 digit
LT	Testo 875-2i thermal camera	Testo AG, Lenzkirch, Germany	−30–350 °C	±2 °C or ±2% of measured value, whichever is greater
LMC	M 5040 P drying oven	Elektromag, İstanbul, Türkiye	5–250 °C	Thermostat accuracy ±1 °C; Chamber temperature variation ±2 °C
	MT3000 precision scale	Knmaster, İstanbul, Türkiye	0.5–3000 g	Resolution: 0.1 g
LPH	PH20S digital pH meter	PCE Instruments, FL, USA	0–14 pH	±0.07 pH (pH 5–9)

T, air temperature; RH, relative humidity; V, air velocity; LT, litter temperature; LMC, litter moisture content; LPH, litter pH.

**Table 2:** Description of the six modeling scenarios, including the predictor variables incorporated in each scenario and the scientific rationale for their inclusion.

Scenario	Predictor Variables	Scientific Purpose
S1	T, RH, Age, Season *	To evaluate baseline predictive performance using environmental and temporal information.
S2	S1 + V	To evaluate the additional contribution of airflow-related information.
S3	S2 + LT	To evaluate the additional predictive contribution of litter thermal conditions.
S4	S3 + BN	To evaluate the additional predictive contribution of flock size.
S5	S4 + X, Y	To evaluate the additional predictive contribution of spatial information within the broiler house.
S6	S5 + LPH (for LMC prediction) S5 + LMC (for LPH prediction)	To evaluate the additional predictive contribution of the complementary litter property under a litter-assisted prediction strategy.

T, air temperature; RH, relative humidity; V, air velocity; LT, litter temperature; BN, bird number; LMC, litter moisture content; LPH, litter pH. * Season was represented using three categorical levels (winter, transition, and summer) and encoded as dummy variables prior to model development.

**Table 3:** Descriptive statistics of the environmental and litter variables measured on Days 7, 21, and 42 of the broiler production cycle.

Day	Variable	N	Min	Max	Mean	SD	Sk	Kr
7	T (°C)	320	27.01	31.84	29.54	0.95	0.10	−0.47
	RH (%)	320	50.76	67.90	58.70	3.13	0.51	0.32
	V (m s^−1^)	320	0.11	0.22	0.17	0.03	0.16	−0.64
	LT (°C)	320	21.90	31.80	27.93	1.80	−0.87	0.97
	LMC (%)	320	15.02	27.71	21.33	3.38	0.27	−1.25
	LPH	320	6.02	7.18	6.52	0.31	0.08	−1.11
21	T (°C)	640	22.58	27.08	25.41	0.78	−0.76	1.43
	RH (%)	640	53.13	79.75	63.26	6.40	0.50	−0.74
	V (m s^−1^)	640	0.15	1.22	0.39	0.18	1.73	3.06
	LT (°C)	640	20.00	32.90	27.08	2.44	−0.43	0.18
	LMC (%)	640	19.09	40.50	31.39	5.16	−0.29	−1.14
	LPH	640	7.17	8.12	7.64	0.18	−0.07	−0.34
42	T (°C)	640	19.00	26.49	21.74	2.36	1.07	−0.68
	RH (%)	640	58.85	79.81	68.80	4.65	0.61	−0.26
	V (m s^−1^)	640	0.20	2.04	0.81	0.49	0.92	−0.65
	LT (°C)	640	24.00	33.40	28.71	2.50	−0.12	−1.21
	LMC (%)	640	25.87	42.88	34.41	4.48	−0.40	−1.12
	LPH	640	7.12	8.34	7.81	0.21	−0.72	0.58

T, air temperature; RH, relative humidity; V, air velocity; LT, litter temperature; LMC, litter moisture content; LPH, litter pH; N, number of observations; SD, standard deviation; Sk, skewness; Kr, kurtosis.

**Table 4:** Held-out outer-test performance of the four ML algorithms for predicting LMC under the six predictor scenarios.

Scenario	Model	R^2^	RMSE	MAE	MBE	MAPE
S1	MLP	0.750 ± 0.095	3.360 ± 0.797	2.727 ± 0.682	−0.394 ± 1.936	9.263 ± 1.511
	RF	0.752 ± 0.185	3.282 ± 1.047	2.629 ± 0.802	0.193 ± 2.014	9.149 ± 2.920
	SVR	0.747 ± 0.129	3.353 ± 1.039	2.618 ± 0.797	−0.047 ± 2.029	8.945 ± 2.201
	XGB	0.737 ± 0.166	3.361 ± 0.893	2.727 ± 0.750	−0.011 ± 2.033	9.438 ± 2.362
S2	MLP	0.738 ± 0.121	3.277 ± 0.971	2.649 ± 0.756	−0.296 ± 1.672	9.013 ± 2.027
	RF	0.778 ± 0.150	3.097 ± 0.859	2.498 ± 0.650	0.083 ± 1.966	8.624 ± 2.389
	SVR	0.754 ± 0.098	3.220 ± 0.757	2.510 ± 0.624	0.177 ± 1.827	8.656 ± 1.894
	XGB	0.786 ± 0.092	3.202 ± 0.844	2.620 ± 0.806	−0.100 ± 2.093	8.925 ± 2.020
S3	MLP	0.764 ± 0.088	3.280 ± 0.960	2.671 ± 0.805	−0.335 ± 2.071	9.028 ± 1.997
	RF	0.759 ± 0.147	3.287 ± 0.909	2.662 ± 0.706	−0.002 ± 2.108	9.082 ± 2.012
	SVR	0.750 ± 0.082	3.295 ± 0.843	2.613 ± 0.691	−0.185 ± 1.990	8.919 ± 1.955
	XGB	0.796 ± 0.118	3.082 ± 0.956	2.503 ± 0.818	−0.008 ± 2.024	8.552 ± 2.508
S4	MLP	0.695 ± 0.183	5.156 ± 2.792	3.995 ± 1.714	−0.709 ± 3.695	14.840 ± 8.272
	RF	0.736 ± 0.156	3.617 ± 0.845	2.940 ± 0.548	0.361 ± 2.405	10.320 ± 2.744
	SVR	0.765 ± 0.142	3.570 ± 1.299	2.666 ± 0.839	−0.203 ± 2.049	9.969 ± 4.002
	XGB	0.793 ± 0.088	3.142 ± 0.854	2.544 ± 0.745	0.001 ± 2.044	8.745 ± 1.889
S5	MLP	0.754 ± 0.119	3.266 ± 1.057	2.601 ± 0.866	0.585 ± 1.966	9.351 ± 3.891
	RF	0.768 ± 0.144	3.384 ± 0.682	2.771 ± 0.537	0.190 ± 2.273	9.531 ± 2.254
	SVR	0.758 ± 0.138	3.243 ± 0.856	2.529 ± 0.731	0.139 ± 1.791	9.123 ± 2.308
	XGB	0.781 ± 0.127	3.023 ± 0.755	2.457 ± 0.638	0.087 ± 1.943	8.356 ± 2.433
S6	MLP	0.649 ± 0.189	3.691 ± 1.421	2.955 ± 1.171	0.697 ± 2.181	10.652 ± 4.864
	RF	0.773 ± 0.166	3.328 ± 1.030	2.715 ± 0.808	0.311 ± 2.396	9.409 ± 3.454
	SVR	0.780 ± 0.124	3.075 ± 0.872	2.433 ± 0.692	0.313 ± 1.873	8.640 ± 2.449
	XGB	0.757 ± 0.174	3.273 ± 1.200	2.604 ± 0.974	0.308 ± 2.305	8.868 ± 3.620

Values are mean ± SD across eight production-period outer-test folds (n = 8 folds; 200 observations per held-out PP). R^2^, coefficient of determination; RMSE, root mean square error; MAE, mean absolute error; MBE, mean bias error; MAPE, mean absolute percentage error; MLP, Multilayer Perceptron; RF, Random Forest; SVR, Support Vector Regression; XGB, Extreme Gradient Boosting.

**Table 5:** Held-out outer-test performance of the four ML algorithms for LPH prediction under the six predictor scenarios.

Scenario	Model	R^2^	RMSE	MAE	MBE	MAPE
S1	MLP	0.858 ± 0.125	0.217 ± 0.086	0.168 ± 0.073	−0.026 ± 0.078	2.304 ± 1.006
	RF	0.868 ± 0.131	0.194 ± 0.048	0.150 ± 0.039	0.003 ± 0.050	2.063 ± 0.561
	SVR	0.869 ± 0.132	0.199 ± 0.072	0.149 ± 0.055	−0.023 ± 0.052	2.067 ± 0.799
	XGB	0.857 ± 0.139	0.203 ± 0.050	0.158 ± 0.041	0.005 ± 0.061	2.177 ± 0.581
S2	MLP	0.878 ± 0.118	0.200 ± 0.055	0.153 ± 0.041	0.009 ± 0.065	2.116 ± 0.615
	RF	0.871 ± 0.124	0.189 ± 0.044	0.147 ± 0.037	0.009 ± 0.045	2.021 ± 0.516
	SVR	0.876 ± 0.124	0.198 ± 0.071	0.150 ± 0.055	−0.028 ± 0.050	2.068 ± 0.803
	XGB	0.870 ± 0.133	0.191 ± 0.053	0.147 ± 0.043	0.012 ± 0.050	2.031 ± 0.616
S3	MLP	0.824 ± 0.267	0.226 ± 0.110	0.169 ± 0.081	−0.034 ± 0.084	2.328 ± 1.096
	RF	0.884 ± 0.123	0.183 ± 0.049	0.147 ± 0.040	0.016 ± 0.058	2.012 ± 0.556
	SVR	0.858 ± 0.105	0.241 ± 0.089	0.188 ± 0.084	−0.032 ± 0.088	2.570 ± 1.169
	XGB	0.865 ± 0.127	0.198 ± 0.054	0.153 ± 0.041	0.017 ± 0.057	2.111 ± 0.600
S4	MLP	0.854 ± 0.134	0.291 ± 0.107	0.212 ± 0.039	−0.093 ± 0.107	2.947 ± 0.632
	RF	0.878 ± 0.120	0.200 ± 0.062	0.157 ± 0.055	0.010 ± 0.052	2.175 ± 0.772
	SVR	0.792 ± 0.269	0.257 ± 0.051	0.191 ± 0.045	0.002 ± 0.114	2.663 ± 0.637
	XGB	0.868 ± 0.131	0.202 ± 0.061	0.156 ± 0.056	−0.010 ± 0.036	2.155 ± 0.779
S5	MLP	0.765 ± 0.330	0.274 ± 0.053	0.194 ± 0.035	−0.024 ± 0.103	2.719 ± 0.494
	RF	0.889 ± 0.109	0.186 ± 0.047	0.145 ± 0.043	0.005 ± 0.054	2.002 ± 0.595
	SVR	0.773 ± 0.324	0.264 ± 0.050	0.188 ± 0.040	0.003 ± 0.090	2.631 ± 0.580
	XGB	0.882 ± 0.119	0.194 ± 0.064	0.150 ± 0.056	0.001 ± 0.058	2.068 ± 0.768
S6	MLP	0.861 ± 0.122	0.246 ± 0.048	0.188 ± 0.030	−0.032 ± 0.126	2.603 ± 0.434
	RF	0.891 ± 0.110	0.181 ± 0.049	0.139 ± 0.040	0.007 ± 0.063	1.922 ± 0.561
	SVR	0.788 ± 0.298	0.256 ± 0.072	0.193 ± 0.061	−0.007 ± 0.107	2.680 ± 0.882
	XGB	0.885 ± 0.115	0.187 ± 0.050	0.147 ± 0.043	0.008 ± 0.064	2.020 ± 0.603

Values are mean ± SD across eight production-period outer-test folds (n = 8 folds; 200 observations per held-out PP). R^2^, coefficient of determination; RMSE, root mean square error; MAE, mean absolute error; MBE, mean bias error; MAPE, mean absolute percentage error; MLP, Multilayer Perceptron; RF, Random Forest; SVR, Support Vector Regression; XGB, Extreme Gradient Boosting.

## Data Availability

The datasets generated and/or analyzed during the current study are available from the corresponding author upon reasonable request.

## Supplementary Materials

The following supporting information can be downloaded at https://www.mdpi.com/article/10.3390/ani16182906/s1. Figure S1: Measured versus predicted LMC values for the RF model under the six predictor scenarios. Outer-training and held-out outer-test results are shown in each panel; Figure S2: Measured versus predicted LMC values for the SVR model under the six predictor scenarios. Outer-training and held-out outer-test results are shown in each panel; Figure S3: Measured versus predicted LMC values for the MLP model under the six predictor scenarios. Outer-training and held-out outer-test results are shown in each panel; Figure S4: Production-period-specific held-out outer-test performance of the RF model for LMC prediction under the six predictor scenarios. Each PP represents one outer LOPO fold; Figure S5: Production-period-specific held-out outer-test performance of the SVR model for LMC prediction under the six predictor scenarios. Each PP represents one outer LOPO fold; Figure S6: Production-period-specific held-out outer-test performance of the MLP model for LMC prediction under the six predictor scenarios. Each PP represents one outer LOPO fold; Figure S7: Measured versus predicted LPH values for the XGB model under the six predictor scenarios. Outer-training and held-out outer-test results are shown in each panel; Figure S8: Measured versus predicted LPH values for the SVR model under the six predictor scenarios. Outer-training and held-out outer-test results are shown in each panel; Figure S9: Measured versus predicted LPH values for the MLP model under the six predictor scenarios. Outer-training and held-out outer-test results are shown in each panel; Figure S10: Production-period-specific held-out outer-test performance of the XGB model for LPH prediction under the six predictor scenarios. Each PP represents one outer LOPO fold; Figure S11: Production-period-specific held-out outer-test performance of the SVR model for LPH prediction under the six predictor scenarios. Each PP represents one outer LOPO fold; Figure S12: Production-period-specific held-out outer-test performance of the MLP model for LPH prediction under the six predictor scenarios. Each PP represents one outer LOPO fold; Table S1: Mathematical expressions and definitions of the performance metrics used for model evaluation; Table S2: Production periods, corresponding dates, seasonal conditions, and number of birds; Table S3: Age-specific descriptive statistics for the complete dataset and the comparable-location subset restricted to the 40 sampling locations represented on days 7, 21, and 42; Table S4: Analytical repeatability of LMC and LPH measurements overall and according to sampling age; Table S5: Production-period-specific descriptive statistics of LMC and LPH across the eight production periods; Table S6: Exploratory assessment of serial dependence across successive production periods; Table S7: Outer-training refit performance of the four ML algorithms for predicting LMC under the six predictor scenarios (mean ± SD); Table S8: Outer-training refit performance of the four ML algorithms for predicting LPH under the six predictor scenarios (mean ± SD); Supplementary Spreadsheet S1: Hyperparameter search ranges and optimal configurations selected for the ML models; Supplementary Spreadsheet S2: Permutation feature importance results for the evaluated ML models; Supplementary Spreadsheet S3: Fold-specific outer-test performance, uncertainty summaries, paired algorithm differences, and pooled out-of-fold performance; Supplementary Analysis Script: Python code used for data preprocessing, nested LOPO-CV model development, hyperparameter optimization, model evaluation, and generation of fold-specific predictions and model outputs.

## References

[1] Papakonstantinou G.I., Voulgarakis N., Terzidou G., Fotos L., Giamouri E., Papatsiros V.G. (2024). Precision livestock farming technology: Applications and challenges of animal welfare and climate change. Agriculture.

[2] Tullo E., Finzi A., Guarino M. (2019). Environmental impact of livestock farming and Precision Livestock Farming as a mitigation strategy. Sci. Total Environ..

[3] Rowe E., Dawkins M.S., Gebhardt-Henrich S.G. (2019). A systematic review of precision livestock farming in the poultry sector: Is technology focussed on improving bird welfare?. Animals.

[4] Mohammadi-Aragh M.K., Norris K.L., Chesser G.D., Lowe J.W., Evans J.D., Purswell J.L., Linhoss J.E. (2025). Comparison of biochar and Poultry Litter Treatment (PLT) amendments on broiler litter quality and bird performance. J. Appl. Poult. Res..

[5] Wilcox C.H., Sandilands V., Mayasari N., Asmara I.Y., Anang A. (2024). A literature review of broiler chicken welfare, husbandry, and assessment. World’s Poult. Sci. J..

[6] Dunlop M.W., McAuley J., Blackall P.J., Stuetz R.M. (2016). Water activity of poultry litter: Relationship to moisture content during a grow-out. J. Environ. Manag..

[7] Swelum A.A., El-Saadony M.T., Abd El-Hack M.E., Ghanima M.M.A., Shukry M., Alhotan R.A., Hussein E.O., Suliman G.M., Ba-Awadh H., Ammari A.A. (2021). Ammonia emissions in poultry houses and microbial nitrification as a promising reduction strategy. Sci. Total Environ..

[8] Miles D., Rowe D., Cathcart T. (2011). High litter moisture content suppresses litter ammonia volatilization. Poult. Sci..

[9] Zuur A.F., Ieno E.N., Walker N.J., Saveliev A.A., Smith G.M. (2009). Mixed Effects Models and Extensions in Ecology with R.

[10] Küçüktopçu E., Cemek B., Simsek H. (2024). Modeling environmental conditions in poultry production: Computational fluid dynamics approach. Animals.

[11] Küçüktopçu E., Cemek B., Yıldırım D., Simsek H., Erensoy K., Sarıca M. (2025). Application of supervised machine learning techniques and digital image analysis for predicting live weight in Anadolu-T broilers. Animals.

[12] Silva N.C.D., Nääs I.D., Barros J.D.G., Moura D.J.D. (2025). Modeling Broiler Discomfort Under Commercial Housing: Seasonal Trends and Predictive Insights for Precision Livestock Farming. Poultry.

[13] Golden C.E., Rothrock M.J., Mishra A. (2019). Comparison between random forest and gradient boosting machine methods for predicting *Listeria* spp. prevalence in the environment of pastured poultry farms. Food Res. Int..

[14] Yuan C., Chen Z.K., Tang Y.R., Zhao R.Q., Liu L.S., Shen M.X. (2025). Caged broiler aggregation behavior recognition via target detection and label merging. Comput. Electron. Agric..

[15] Rawdhan S.A., Abdelhamid M.A., Anwer A.A., Shaaban F.M. (2026). An IoT and Machine Learning Approach for Predicting Air Quality Parameters in Poultry Houses. J. Biosyst. Eng..

[16] Liu Y., Zhuang Y., Ji B., Zhang G., Rong L., Teng G., Wang C. (2022). Prediction of laying hen house odor concentrations using machine learning models based on small sample data. Comput. Electron. Agric..

[17] Küçüktopçu E., Cemek B., Simsek H. (2024). Machine learning and wavelet transform: A hybrid approach to predicting ammonia levels in poultry farms. Animals.

[18] Miles D.M., Rowe D.E., Brooks J.P. (2017). Implications of intensive spatial sampling of broiler litter: Characteristics and gaseous emissions. Int. J. Poult. Sci..

[19] Tasistro A.S., Kissel D.E., Bush P.B. (2004). Spatial variability of broiler litter composition in a chicken house. J. Appl. Poult. Res..

[20] Miles D., Rowe D., Cathcart T. (2011). Litter ammonia generation: Moisture content and organic versus inorganic bedding materials. Poult. Sci..

[21] Qasim W., Moon B.E., Phonsuwan M., Jo J.S., Lee M.H., Nafees M., Kim H.T. (2018). Effects of an aluminum sulfate and ferric chloride blend on poultry litter characteristics in vitro. J. Appl. Poult. Res..

[22] Pirompud P., Sivapirunthep P., Punyapornwithaya V., Chaosap C. (2024). Application of machine learning algorithms to predict dead on arrival of broiler chickens raised without antibiotic program. Poult. Sci..

[23] Liakos K.G., Busato P., Moshou D., Pearson S., Bochtis D. (2018). Machine learning in agriculture: A review. Sensors.

[24] Coelho Ribeiro L.A., Bresolin T., Rosa G.J.d.M., Rume Casagrande D., Danes M.d.A.C., Dórea J.R.R. (2021). Disentangling data dependency using cross-validation strategies to evaluate prediction quality of cattle grazing activities using machine learning algorithms and wearable sensor data. J. Anim. Sci..

[25] Manav H.R., Narinç D., Aygun A., Öksüz Narinç N., Kaya Başar E., Fırat M.Z. (2026). Bayesian Multi-Model Comparison and Nonlinear Mixed Modelling of Growth Trajectories in Denizli Chickens. Animals.

[26] Xu Y., Teng G., Zhou Z. (2024). Short-term prediction method for gas concentration in poultry houses under different feeding patterns. Agriculture.

[27] Tulsi A., Momin A., Ayres V. (2025). Moisture prediction in chicken litter using hyperspectral data and machine learning. Smart Agric. Technol..

[28] Karlsson A., Wang T., Nowaczyk S., Pashami S., Asadi S. (2024). Mind the Data, Measuring the Performance Gap Between Tree Ensembles and Deep Learning on Tabular Data. International Symposium on Intelligent Data Analysis.

[29] Borisov V., Leemann T., Seßler K., Haug J., Pawelczyk M., Kasneci G. (2022). Deep neural networks and tabular data: A survey. IEEE Trans. Neural Netw. Learn. Syst..

[30] Dunlop M.W., Blackall P.J., Stuetz R.M. (2015). Water addition, evaporation and water holding capacity of poultry litter. Sci. Total Environ..

[31] Liu Z., Wang L., Beasley D., Oviedo E. (2007). Effect of moisture content on ammonia emissions from broiler litter: A laboratory study. J. Atmos. Chem..

[32] Miles D.M., Brooks J.P., Moore P.A. (2016). Mid-flock and post-harvest spatial characterization of broiler litter gas flux and nutrients. Int. J. Poult. Sci..

