# Supplementary material for: Machine Learning Prediction of Litter Moisture and Litter pH in a Commercial Broiler House Using Nested Leave-One Production-Period-Out Cross-Validation
Source: Animals (Basel). 2026 Sep 15;16(18):2906. doi: 10.3390/ani16182906 (PMC13603877; doi:10.3390/ani16182906)
Supplement: Supplementary file 1 [file animals-16-02906-s001.zip › Supplemantary_Figure.pdf]

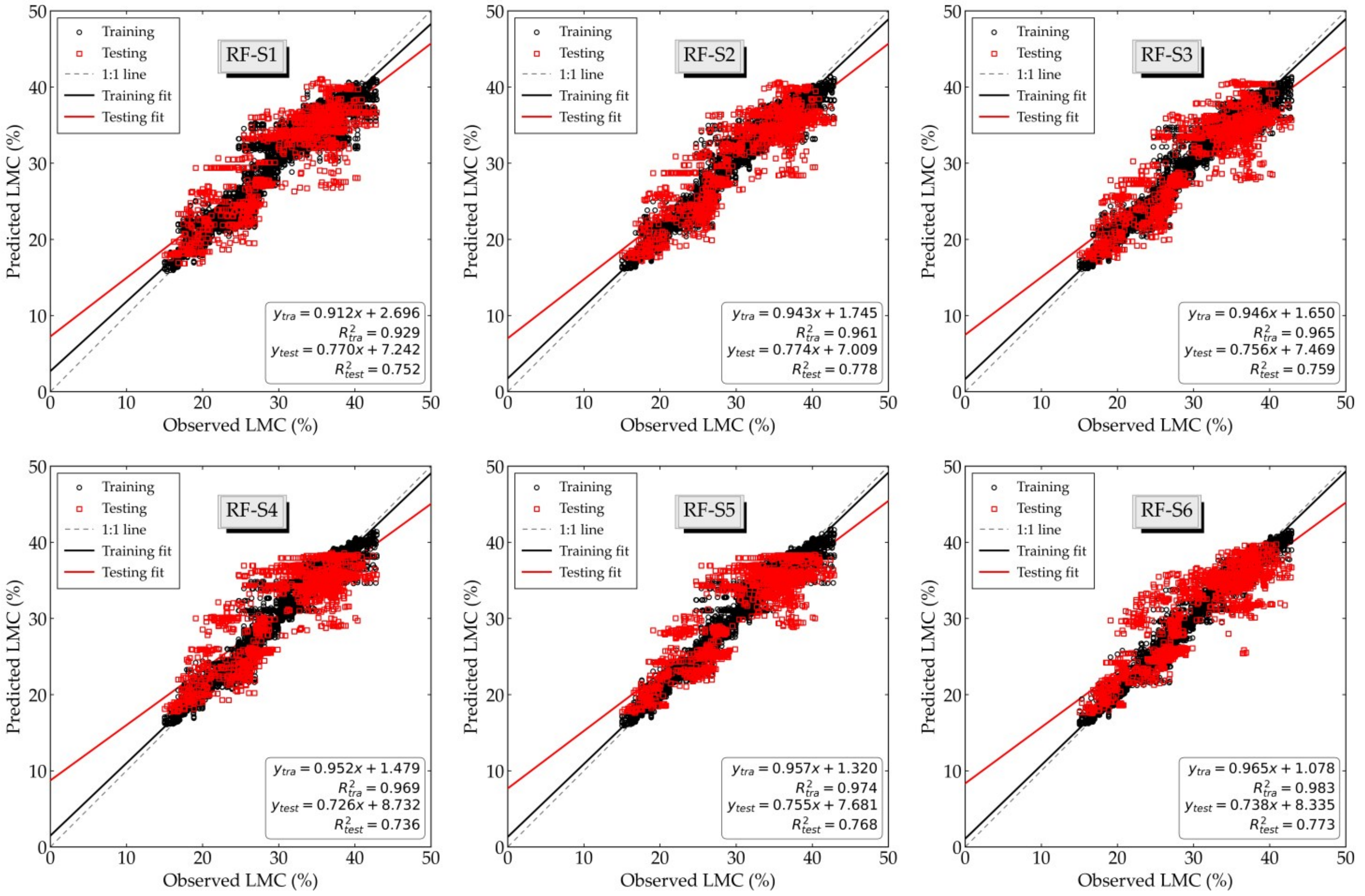

Figure S1. Measured versus predicted LMC values for the RF model under the six predictor scenarios. Outer-training and held-out outer-test results are shown in each panel. *(LMC, litter moisture content; RF, Random Forest).*

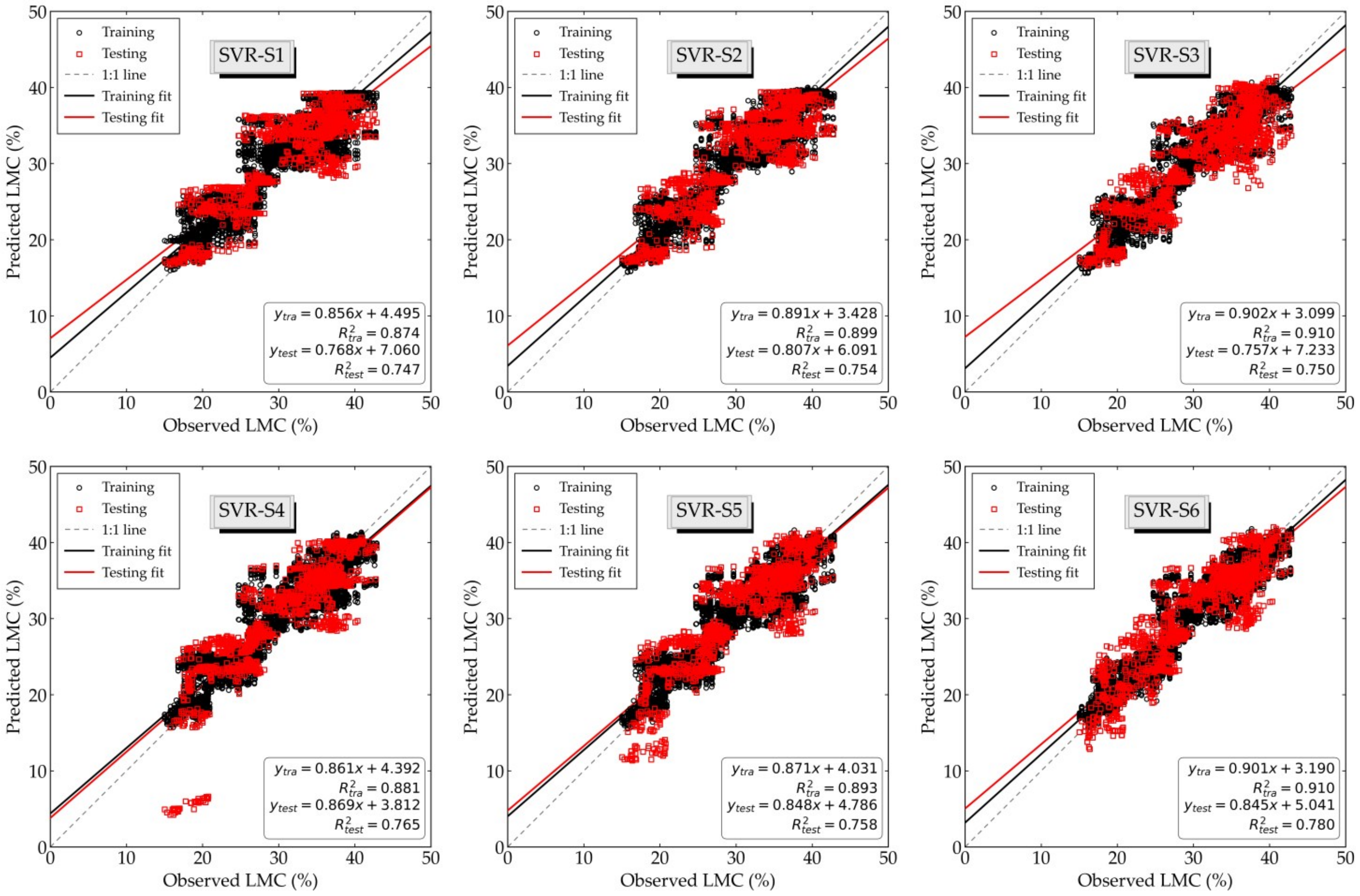

Figure S2. Measured versus predicted LMC values for the SVR model under the six predictor scenarios. Outer-training and held-out outer-test results are shown in each panel. *(LMC, litter moisture content; SVR, Support Vector Regression).*

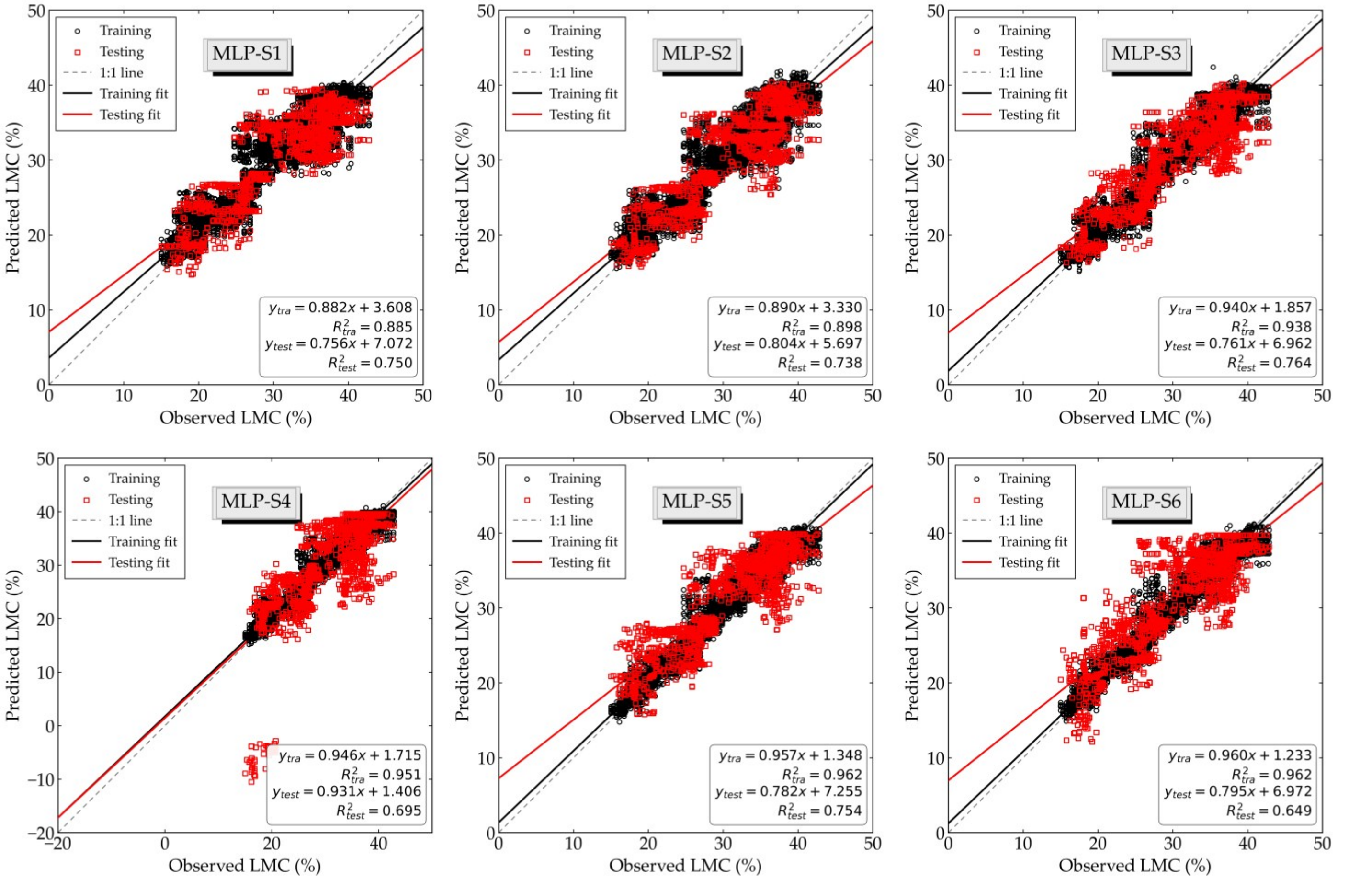

Figure S3. Measured versus predicted LMC values for the MLP model under the six predictor scenarios. Outer-training and held-out outer-test results are shown in each panel. *(LMC, litter moisture content; MLP, Multilayer Perceptron).*

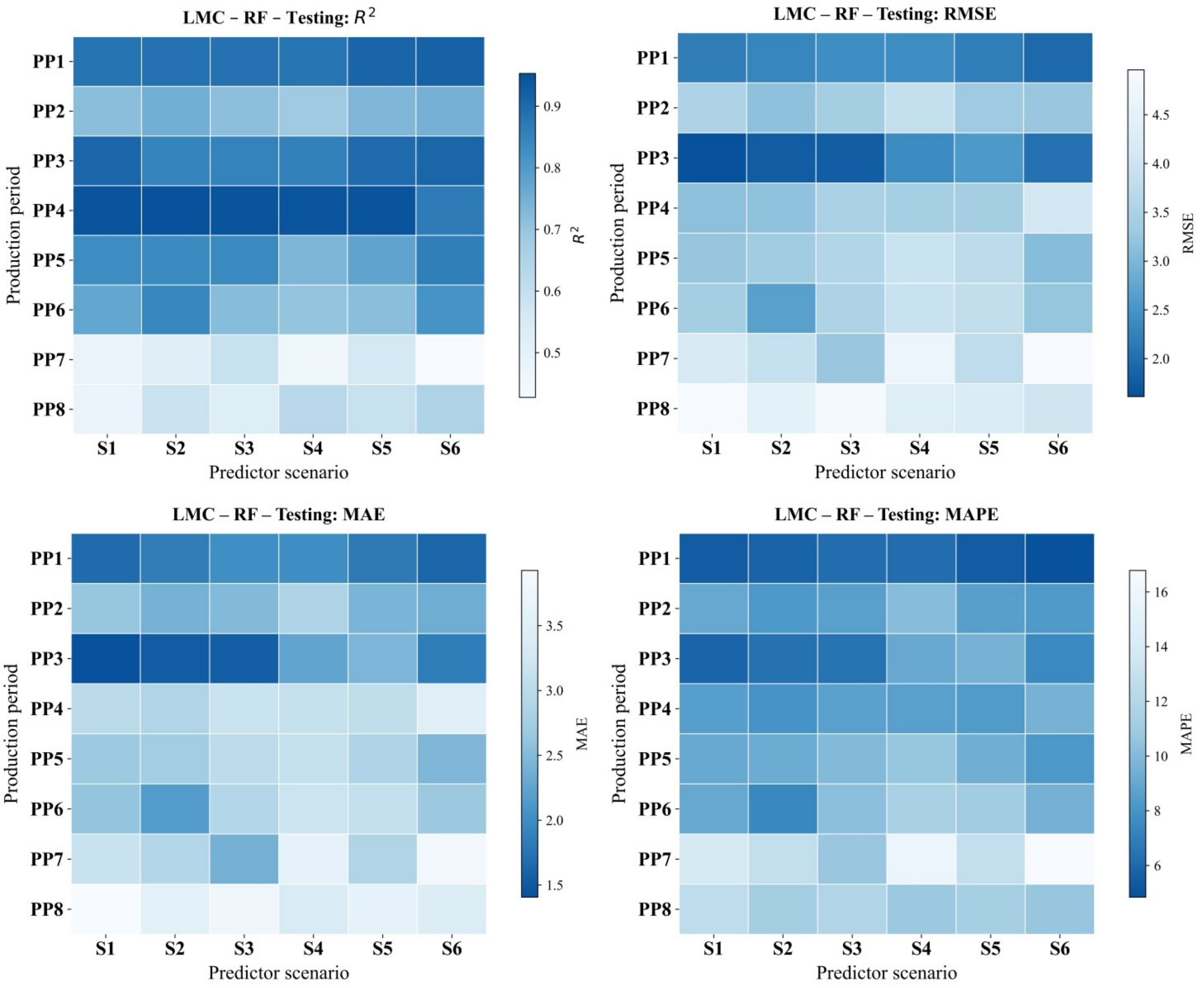

Figure S4. Production-period-specific held-out outer-test performance of the RF model for LMC prediction under the six predictor scenarios. Each PP represents one outer LOPO fold. *(LMC, litter moisture content; RF, Random Forest; PP, production period; LOPO, Leave-One-Production-Period-Out; R^2, coefficient of determination; RMSE, root mean square error; MAE, mean absolute error; MAPE, mean absolute percentage error).*

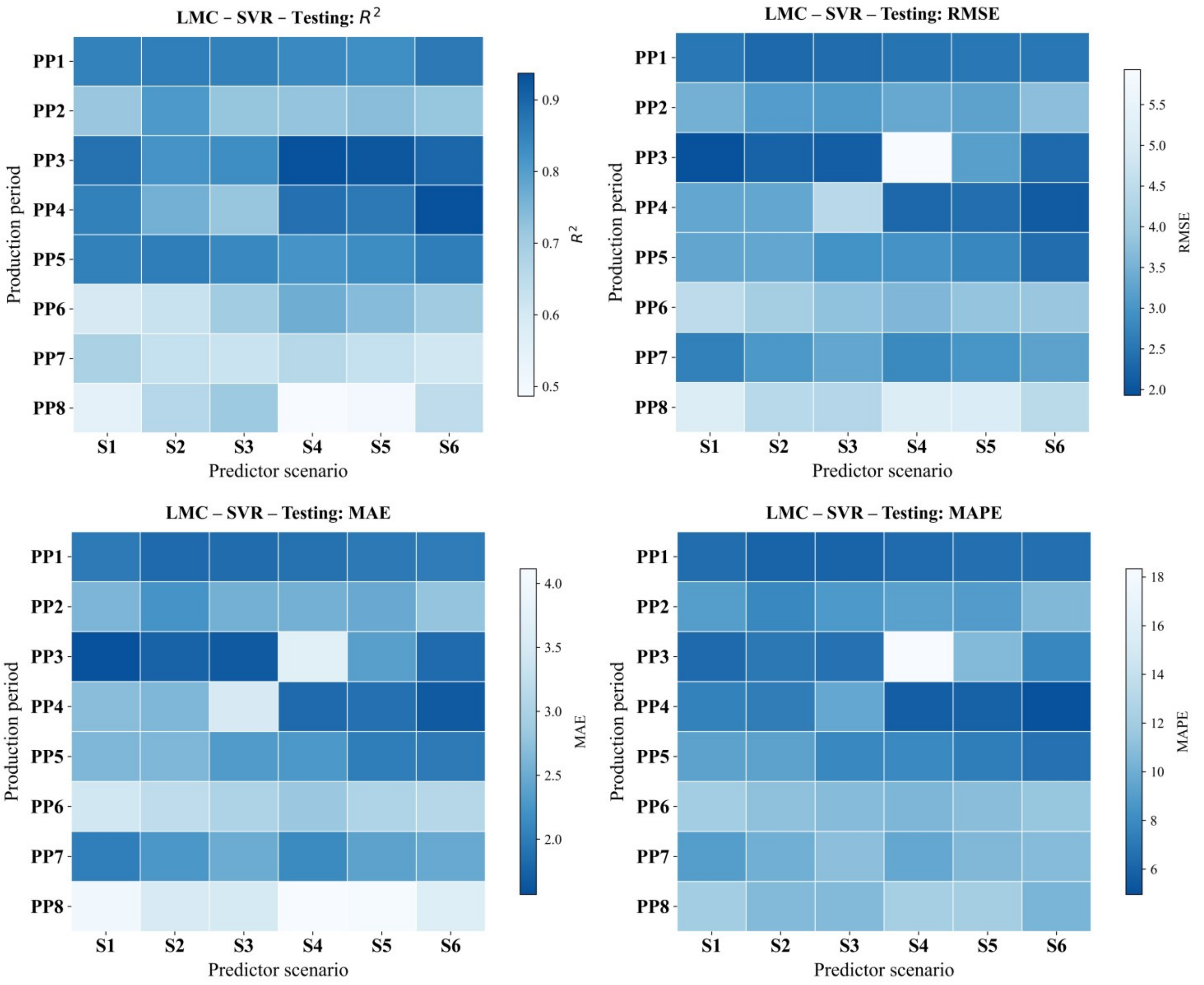

Figure S5. Production-period-specific held-out outer-test performance of the SVR model for LMC prediction under the six predictor scenarios. Each PP represents one outer LOPO fold. *(LMC, litter moisture content; SVR, Support Vector Regression; PP, production period; LOPO, Leave-One-Production-Period-Out; R^2, coefficient of determination; RMSE, root mean square error; MAE, mean absolute error; MAPE, mean absolute percentage error).*

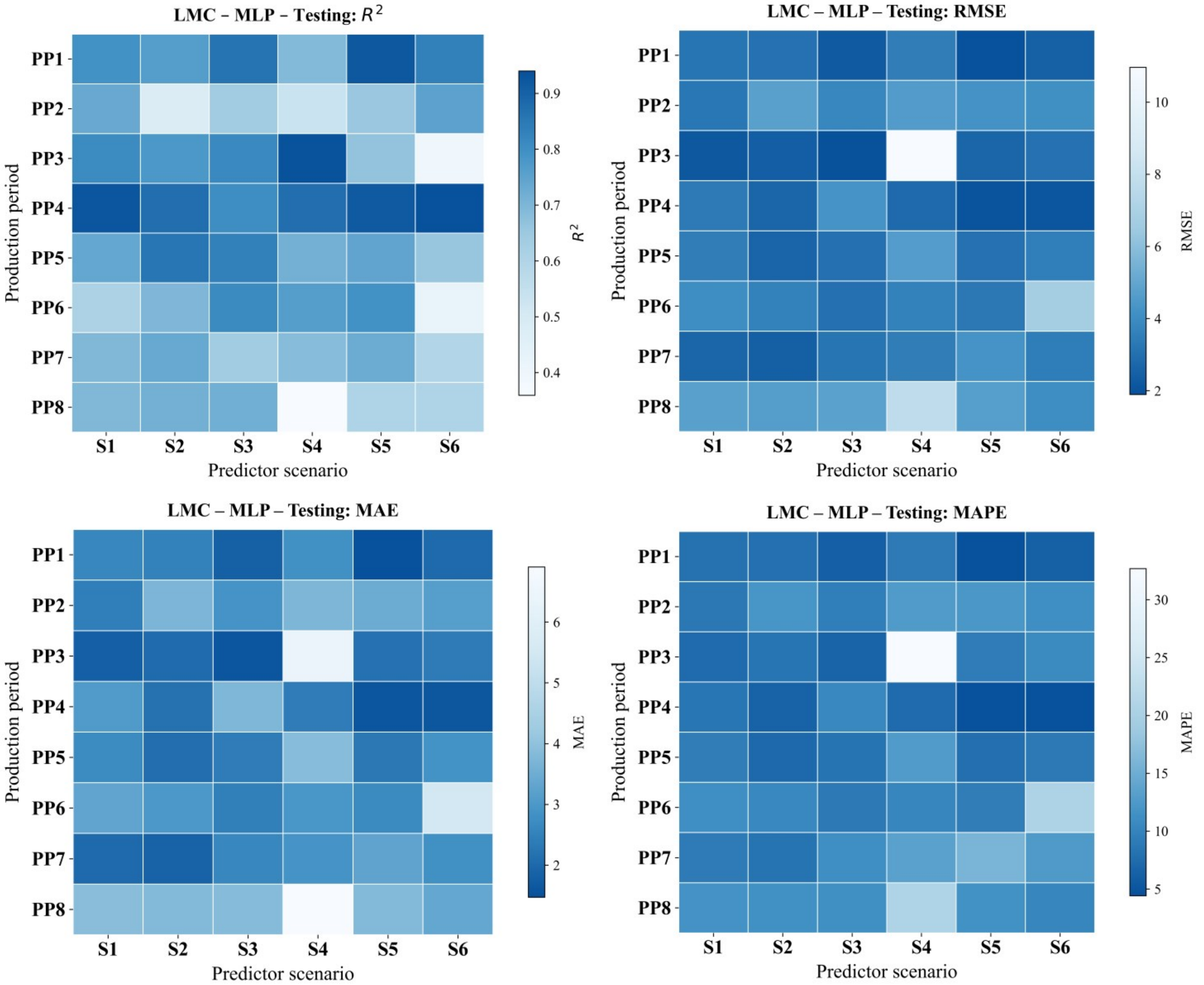

Figure S6. Production-period-specific held-out outer-test performance of the MLP model for LMC prediction under the six predictor scenarios. Each PP represents one outer LOPO fold. *(LMC, litter moisture content; MLP, Multilayer Perceptron; PP, production period; LOPO, Leave-One-Production-Period-Out;* R^2*, coefficient of determination; RMSE, root mean square error; MAE, mean absolute error; MAPE, mean absolute percentage error).*

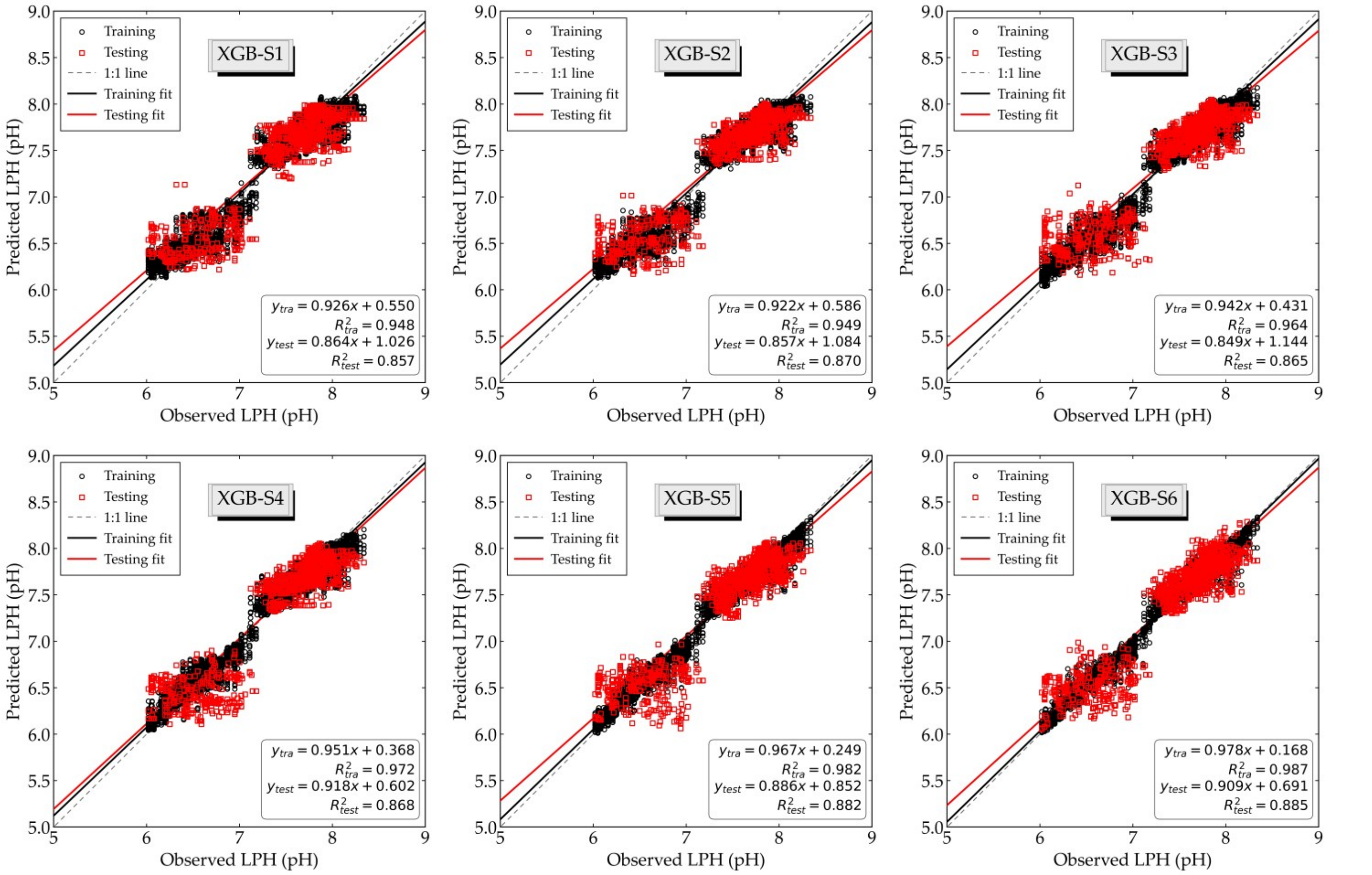

Figure S7. Measured versus predicted LPH values for the XGB model under the six predictor scenarios. Outer-training and held-out outer-test results are shown in each panel. *(LPH, litter pH; XGB, Extreme Gradient Boosting).*

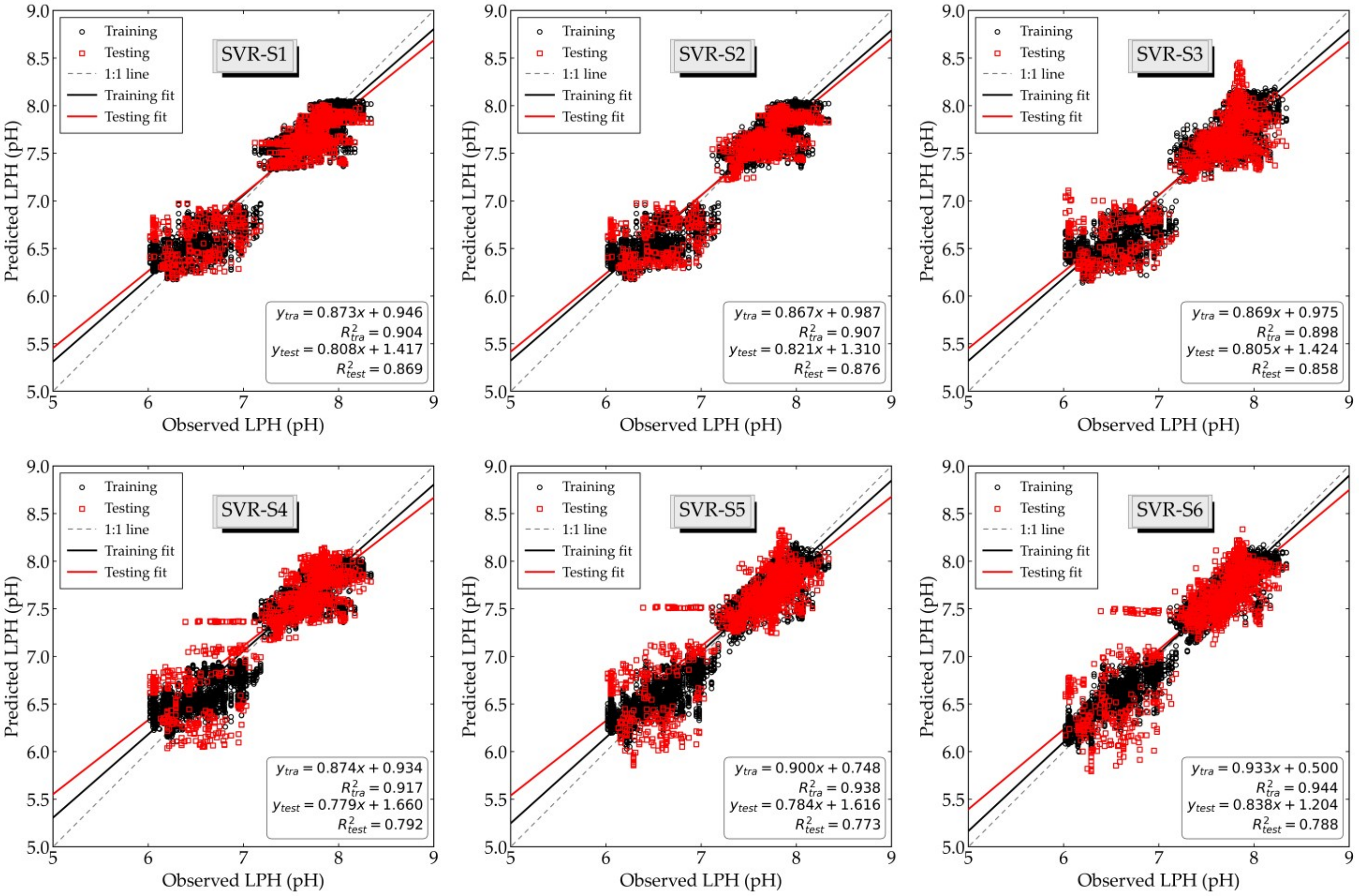

Figure S8. Measured versus predicted LPH values for the SVR model under the six predictor scenarios. Outer-training and held-out outer-test results are shown in each panel. *(LPH, litter pH; SVR, Support Vector Regression).*

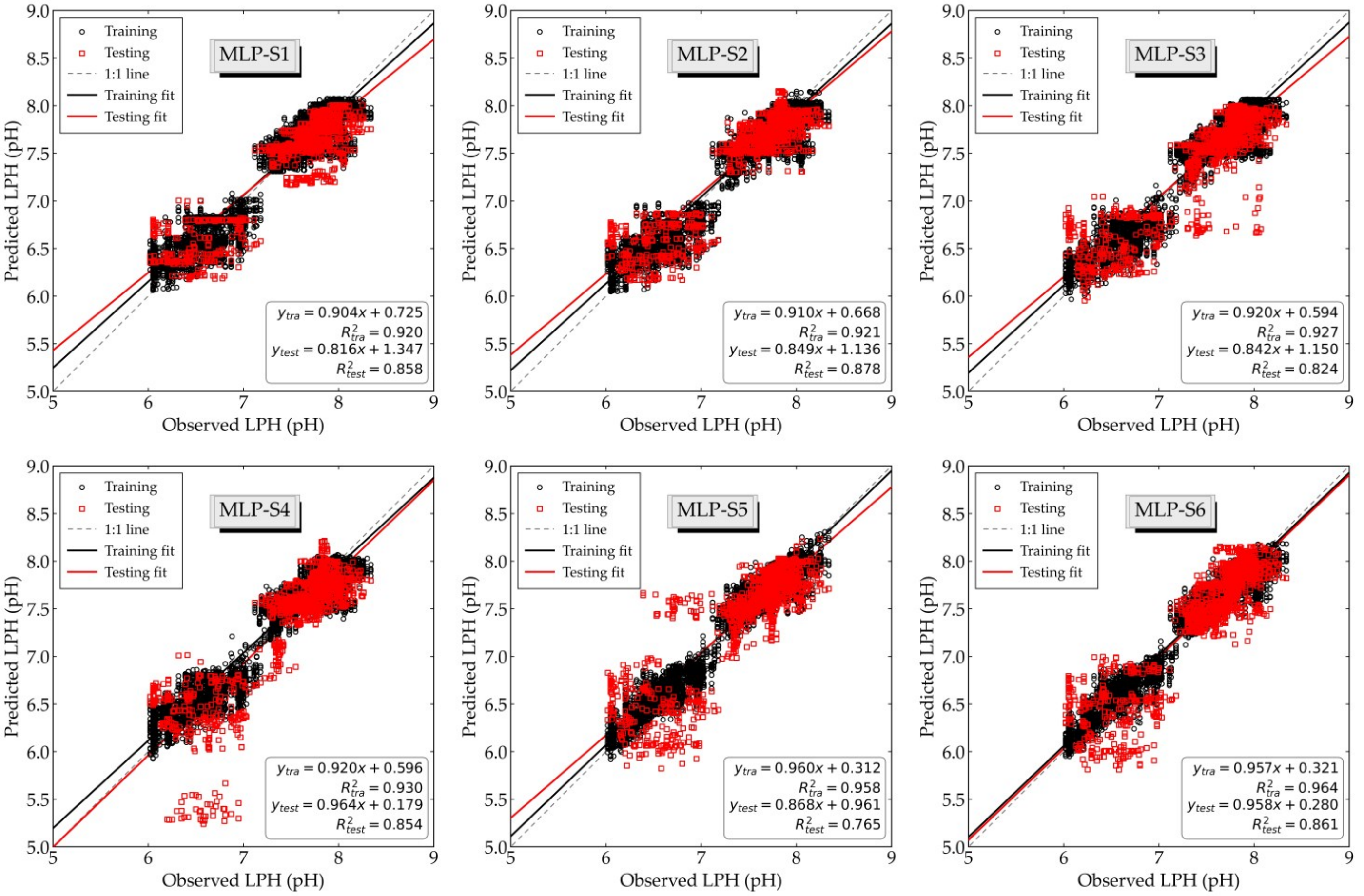

Figure S9. Measured versus predicted LPH values for the MLP model under the six predictor scenarios. Outer-training and held-out outer-test results are shown in each panel. *(LPH, litter pH; MLP, Multilayer Perceptron).*

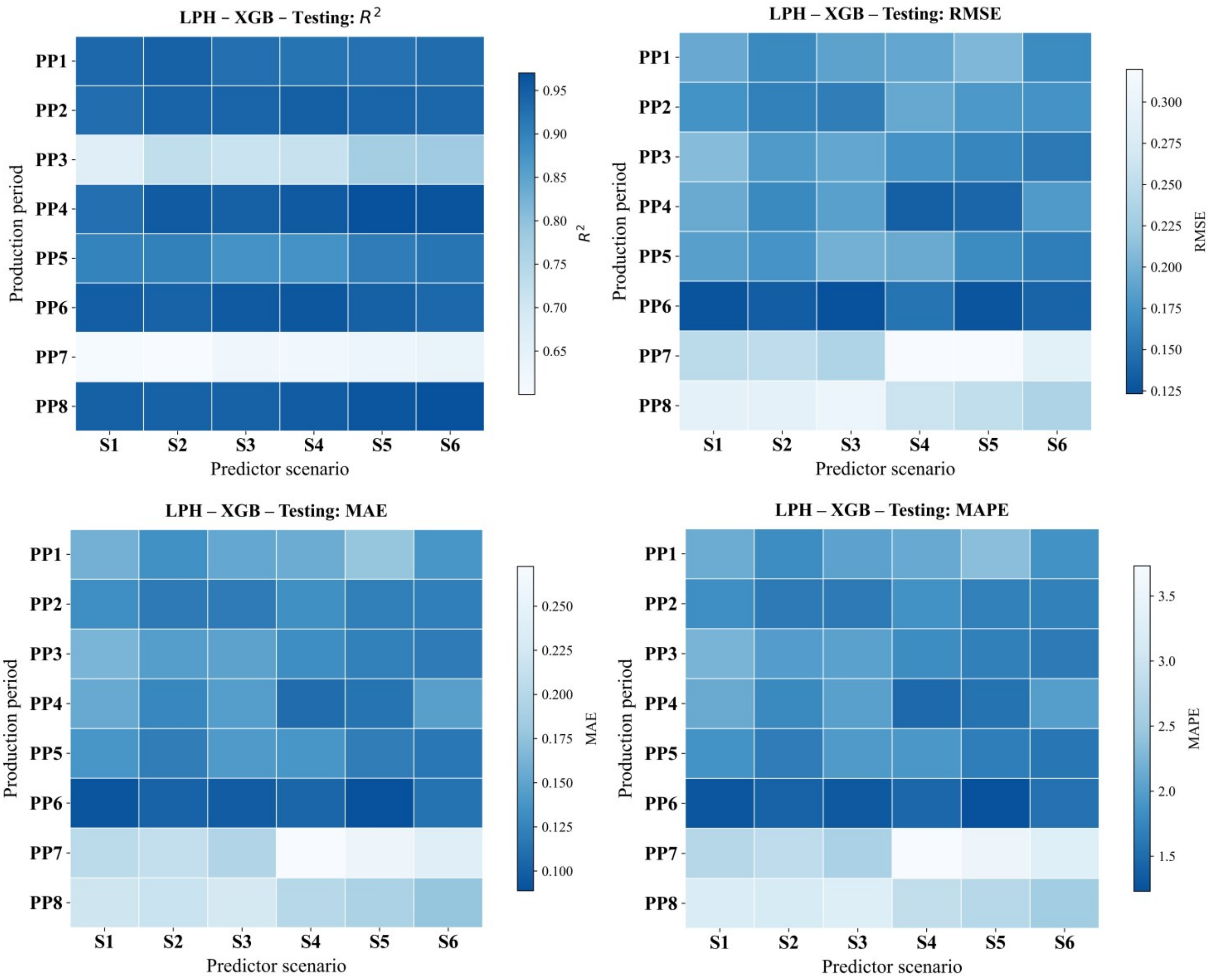

Figure S10. Production-period-specific held-out outer-test performance of the XGB model for LPH prediction under the six predictor scenarios. Each PP represents one outer LOPO fold. *(LPH, litter pH; XGB, Extreme Gradient Boosting; PP, production period; LOPO, Leave-One-Production-Period-Out; R^2, coefficient of determination; RMSE, root mean square error; MAE, mean absolute error; MAPE, mean absolute percentage error).*

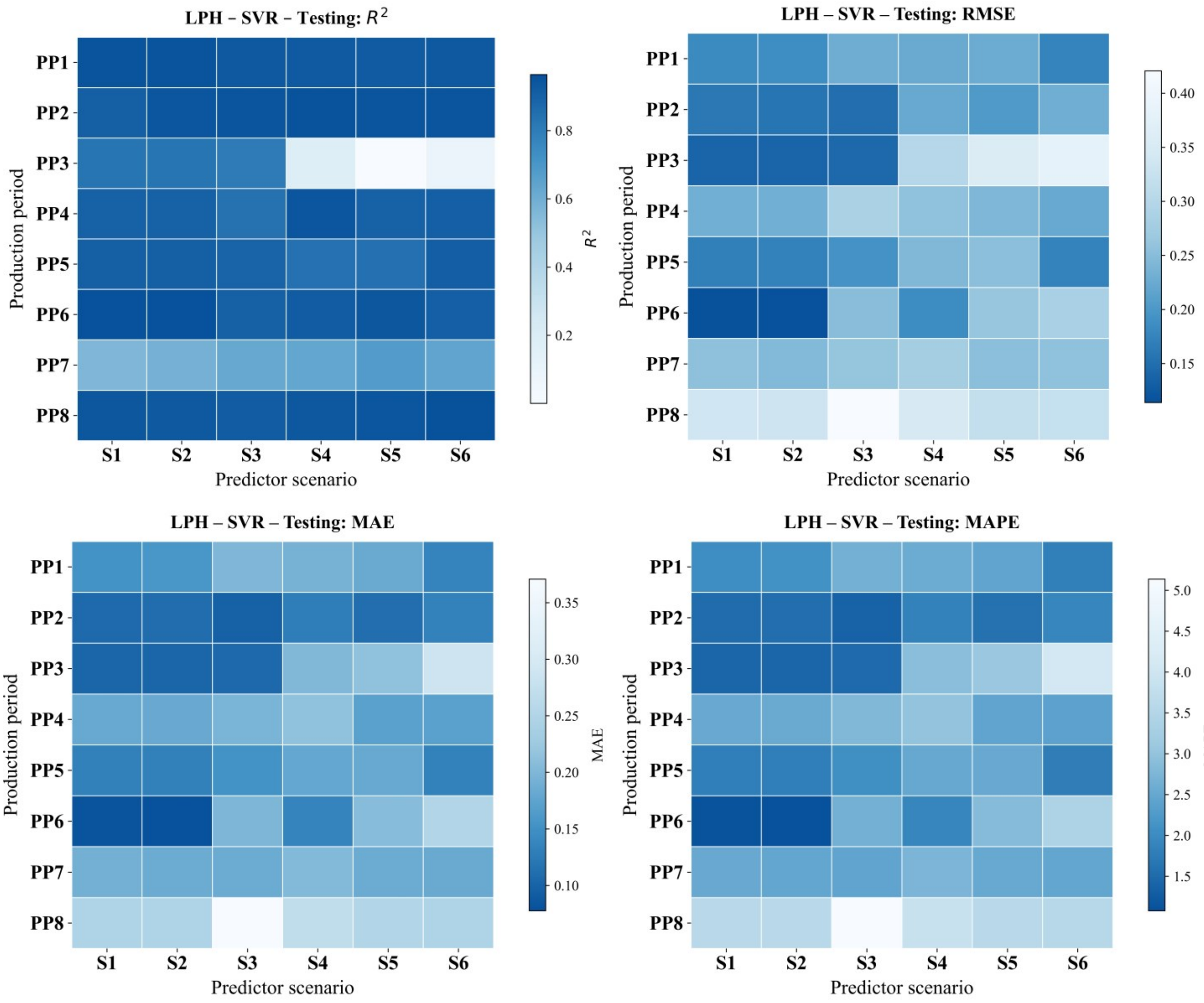

Figure S11. Production-period-specific held-out outer-test performance of the SVR model for LPH prediction under the six predictor scenarios. Each PP represents one outer LOPO fold. *(LPH, litter pH; SVR, Support Vector Regression; PP, production period; LOPO, Leave-One-Production-Period-Out; R^2, coefficient of determination; RMSE, root mean square error; MAE, mean absolute error; MAPE, mean absolute percentage error).*

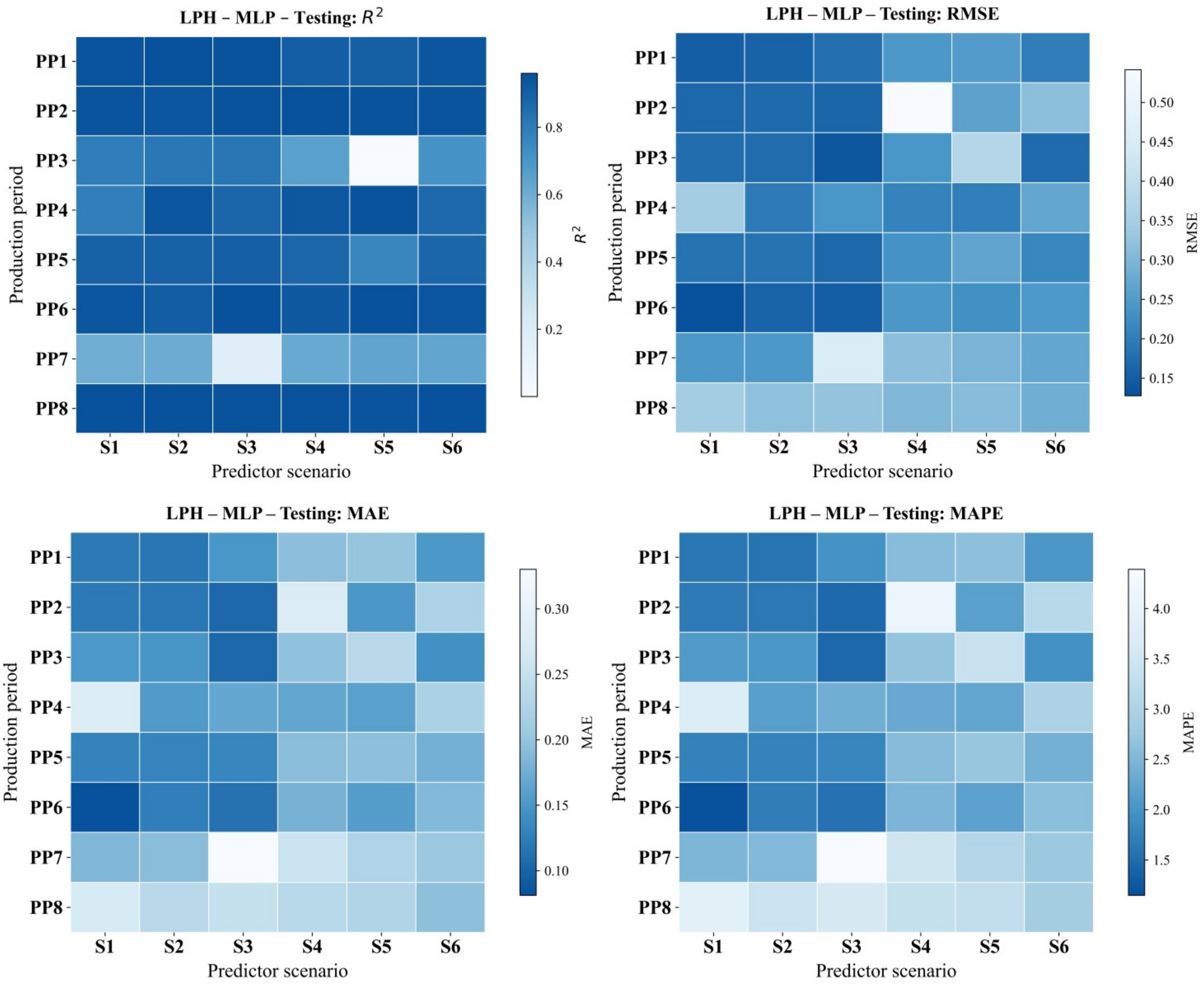

Figure S12. Production-period-specific held-out outer-test performance of the MLP model for LPH prediction under the six predictor scenarios. Each PP represents one outer LOPO fold. *(LPH, litter pH; MLP, Multilayer Perceptron; PP, production period; LOPO, Leave-One-Production-Period-Out; R^2, coefficient of determination; RMSE, root mean square error; MAE, mean absolute error; MAPE, mean absolute percentage error).*
