# Supplementary material for: Machine Learning Prediction of Litter Moisture and Litter pH in a Commercial Broiler House Using Nested Leave-One Production-Period-Out Cross-Validation
Source: Animals (Basel). 2026 Sep 15;16(18):2906. doi: 10.3390/ani16182906 (PMC13603877; doi:10.3390/ani16182906)
Supplement: Supplementary file 1 [file animals-16-02906-s001.zip › Supplemantary_Table.pdf]

Table S1. Mathematical expressions and definitions of the performance metrics used for model evaluation.

Coefficient of Determination (R^2)	$R^2 = 1 - \frac{\sum_{i=1}^{n}(y_i - \hat{y}_i)^2}{\sum_{i=1}^{n}(y_i - \bar{y})^2}$
Root Mean Square Error (RMSE)	$RMSE = \sqrt{\frac{1}{n}\sum_{i=1}^{n}(y_i - \hat{y}_i)^2}$
Mean Absolute Error (MAE)	$MAE = \frac{1}{n}\sum_{i=1}^{n} \mid y_i - \hat{y}_i \mid$
Mean Absolute Percentage Error (MAPE)	$MAPE = \frac{100}{n}\sum_{i=1}^{n} \mid \frac{y_i - \hat{y}_i}{y_i} \mid$
Mean Bias Error (MBE)	$MBE = \frac{1}{n}\sum_{i=1}^{n}(\hat{y}_i - y_i)$

where: $\boldsymbol{y_i}$and $\boldsymbol{\hat{y}_i}$ denote the observed and predicted values for observation $\boldsymbol{i}$, respectively, and $\boldsymbol{n}$ denotes the number of observations in the evaluation set. For MAPE, observations with $\boldsymbol{y_i = 0}$ were excluded from the percentage-error calculation because the denominator is undefined at zero; if no non-zero observed values were available, MAPE was considered undefined. No zero values occurred for either LMC or LPH in the present dataset. Metrics were first calculated across all observations within each corresponding training or held-out outer-test dataset. Outer-test performance reported as mean ± SD was then summarized across the eight production-period-specific outer folds.

Table S2. Production periods, corresponding dates, seasonal conditions, and number of birds.

Production period	Dates	Season	Number of birds
PP1	03 Feb 2018–16 Mar 2018	Winter	20,035
PP2	09 Apr 2018–20 May 2018	Transition	19,840
PP3	12 Jun 2018–22 Jul 2018	Summer	24,000
PP4	09 Nov 2018–19 Dec 2018	Winter	19,440
PP5	09 Jan 2019–18 Feb 2019	Winter	17,760
PP6	14 Mar 2019–24 Apr 2019	Transition	18,000
PP7	16 Jul 2019–26 Aug 2019	Summer	18,240
PP8	11 Sep 2019–23 Oct 2019	Transition	18,384

PP, production period. Seasonal classification was based on the production period in which each flock was raised.

Table S3. Age-specific descriptive statistics for the complete dataset and the comparable-location subset restricted to the 40 sampling locations represented on days 7, 21, and 42.

Day	Dataset	Variable	N	Mean	SD	Median	IQR	Min	Max
7	Full	T (°C)	320	29.54	0.95	29.56	1.32	27.01	31.84
7	Full	RH (%)	320	58.70	3.13	57.92	4.50	50.76	67.90
7	Full	V (m s^{-1})	320	0.166	0.025	0.160	0.030	0.110	0.220
7	Full	LT (°C)	320	27.93	1.80	28.20	2.22	21.90	31.80
7	Full	LMC (%)	320	21.33	3.38	20.57	6.33	15.02	27.71
7	Full	LPH	320	6.517	0.307	6.520	0.490	6.020	7.180
7	Common	T (°C)	320	29.54	0.95	29.56	1.32	27.01	31.84
7	Common	RH (%)	320	58.70	3.13	57.92	4.50	50.76	67.90
7	Common	V (m s^{-1})	320	0.166	0.025	0.160	0.030	0.110	0.220
7	Common	LT (°C)	320	27.93	1.80	28.20	2.22	21.90	31.80
7	Common	LMC (%)	320	21.33	3.38	20.57	6.33	15.02	27.71
7	Common	LPH	320	6.517	0.307	6.520	0.490	6.020	7.180
21	Full	T (°C)	640	25.41	0.78	25.50	0.91	22.58	27.08
21	Full	RH (%)	640	63.26	6.40	62.79	9.93	53.13	79.75
21	Full	V (m s^{-1})	640	0.391	0.185	0.320	0.120	0.150	1.220
21	Full	LT (°C)	640	27.08	2.44	27.30	3.02	20.00	32.90
21	Full	LMC (%)	640	31.39	5.16	32.22	9.73	19.09	40.50
21	Full	LPH	640	7.640	0.181	7.670	0.230	7.170	8.120
21	Common	T (°C)	320	25.21	0.83	25.34	0.89	22.58	26.84
21	Common	RH (%)	320	63.11	6.08	63.16	10.91	53.13	76.07
21	Common	V (m s^{-1})	320	0.344	0.142	0.300	0.122	0.150	0.770
21	Common	LT (°C)	320	27.02	2.68	27.20	3.20	20.00	32.90
21	Common	LMC (%)	320	31.04	5.23	31.63	10.09	20.50	39.33
21	Common	LPH	320	7.644	0.167	7.670	0.200	7.170	8.070
42	Full	T (°C)	640	21.74	2.36	20.57	2.38	19.00	26.49
42	Full	RH (%)	640	68.80	4.65	67.81	6.26	58.85	79.81
42	Full	V (m s^{-1})	640	0.807	0.485	0.600	0.660	0.200	2.040
42	Full	LT (°C)	640	28.71	2.50	28.50	3.90	24.00	33.40
42	Full	LMC (%)	640	34.41	4.48	35.72	8.89	25.87	42.88
42	Full	LPH	640	7.810	0.210	7.840	0.210	7.120	8.340
42	Common	T (°C)	320	21.69	2.27	20.51	2.23	19.23	25.89
42	Common	RH (%)	320	69.27	4.69	68.39	5.42	59.30	79.81
42	Common	V (m s^{-1})	320	0.722	0.467	0.540	0.447	0.200	2.040
42	Common	LT (°C)	320	28.73	2.61	28.40	4.30	24.00	33.40
42	Common	LMC (%)	320	34.25	4.60	35.02	9.29	26.35	42.76
42	Common	LPH	320	7.801	0.192	7.830	0.200	7.260	8.240

Full, complete age-specific dataset; Common, comparable-location subset restricted to the 40 sampling locations represented on days 7, 21, and 42 (Y = 3, 15, 27, and 39 m). T, air temperature; RH, relative humidity; V, air velocity; LT, litter temperature; LMC, litter moisture content; LPH, litter pH; SD, standard deviation; IQR, interquartile range. The complete dataset contained 320 observations on day 7 and 640 observations on days 21 and 42. The comparable-location subset contained 320 observations at each age, yielding 960 observations in total.

Table S4. Analytical repeatability of LMC and LPH measurements overall and according to sampling age.

Response variable	Sampling age	N locations	Mean	Pooled within-location SD	Pooled CV (%)	Repeatability-related standard uncertainty of triplicate mean
LMC	Overall	1600	30.59	0.146	0.476	0.084
LMC	Day 7	320	21.33	0.144	0.673	0.083
LMC	Day 21	640	31.39	0.145	0.458	0.084
LMC	Day 42	640	34.42	0.149	0.432	0.086
LPH	Overall	1600	7.48	0.0287	0.383	0.0166
LPH	Day 7	320	6.52	0.0295	0.453	0.0170
LPH	Day 21	640	7.64	0.0288	0.376	0.0166
LPH	Day 42	640	7.81	0.0281	0.360	0.0163

Table S5. Production-period-specific descriptive statistics of LMC and LPH across the eight production periods.

Period	Season	Variable	N	Mean	SD	Median	IQR	Minimum	Maximum
PP1	Winter	LMC	200	34.08	5.86	36.12	4.29	16.79	42.60
PP1	Winter	LPH	200	7.602	0.577	7.760	0.283	6.13	8.26
PP2	Transition	LMC	200	30.45	5.80	32.42	7.19	17.21	37.94
PP2	Transition	LPH	200	7.472	0.471	7.605	0.323	6.20	7.98
PP3	Summer	LMC	200	25.31	3.86	26.83	2.77	15.02	29.87
PP3	Summer	LPH	200	7.324	0.317	7.390	0.155	6.39	7.96
PP4	Winter	LMC	200	35.71	5.95	37.85	2.55	20.20	42.88
PP4	Winter	LPH	200	7.495	0.629	7.730	0.323	6.02	8.23
PP5	Winter	LMC	200	32.21	5.59	32.86	7.85	17.46	40.88
PP5	Winter	LPH	200	7.562	0.546	7.760	0.275	6.02	8.26
PP6	Transition	LMC	200	29.96	5.85	30.51	9.40	18.01	38.59
PP6	Transition	LPH	200	7.512	0.511	7.700	0.223	6.29	7.99
PP7	Summer	LMC	200	23.97	3.56	23.88	5.96	15.79	29.51
PP7	Summer	LPH	200	7.396	0.381	7.400	0.353	6.42	8.17
PP8	Transition	LMC	200	33.00	6.26	34.86	3.51	18.49	42.76
PP8	Transition	LPH	200	7.506	0.717	7.805	0.300	6.05	8.34

LMC, litter moisture content; LPH, litter pH; SD, standard deviation; IQR, interquartile range; PP, production period. Each production period comprised 200 observations. Values were calculated separately for each production period to characterize between-period heterogeneity.

Table S6. Exploratory assessment of serial dependence across successive production periods.

Variable	Lag-1 Pearson *r*	*P*	Lag-1 Spearman ρ	*P*	Durbin–Watson
LMC	−0.299	0.514	−0.250	0.589	2.375
LPH	−0.065	0.890	0.000	1.000	1.831
T	−0.246	0.594	0.000	1.000	2.355
RH	−0.074	0.875	0.214	0.645	2.034
V	−0.331	0.469	−0.214	0.645	2.410
LT	0.002	0.996	0.000	1.000	1.912

LMC, litter moisture content; LPH, litter pH; T, air temperature; RH, relative humidity; V, air velocity; LT, litter surface temperature. Analyses were based on eight chronologically ordered production-period means, yielding seven successive lag-1 pairs. Because only eight production periods were available, these analyses were exploratory and were not used to establish statistical independence.

Table S7. Outer-training refit performance of the four ML algorithms for predicting LMC under the six predictor scenarios (mean ± SD).

Scenario	Model	R^2	RMSE	MAE	MBE	MAPE
S1	MLP	0.885 ± 0.035	2.221 ± 0.313	1.611 ± 0.258	0.003 ± 0.054	5.599 ± 0.984
	RF	0.929 ± 0.047	1.671 ± 0.595	1.186 ± 0.475	−0.000 ± 0.003	4.016 ± 1.605
	SVR	0.874 ± 0.031	2.349 ± 0.300	1.629 ± 0.260	0.081 ± 0.080	5.732 ± 0.978
	XGB	0.919 ± 0.020	1.882 ± 0.248	1.363 ± 0.193	0.000 ± 0.001	4.668 ± 0.668
S2	MLP	0.898 ± 0.055	2.058 ± 0.537	1.525 ± 0.427	−0.048 ± 0.074	5.340 ± 1.543
	RF	0.961 ± 0.041	1.179 ± 0.588	0.837 ± 0.476	−0.001 ± 0.002	2.865 ± 1.592
	SVR	0.899 ± 0.032	2.086 ± 0.335	1.430 ± 0.269	0.096 ± 0.078	5.014 ± 0.926
	XGB	0.976 ± 0.020	0.973 ± 0.340	0.690 ± 0.262	0.000 ± 0.001	2.369 ± 0.919
S3	MLP	0.938 ± 0.017	1.640 ± 0.223	1.181 ± 0.179	0.007 ± 0.083	4.120 ± 0.640
	RF	0.965 ± 0.037	1.133 ± 0.563	0.802 ± 0.449	0.000 ± 0.003	2.753 ± 1.471
	SVR	0.910 ± 0.036	1.961 ± 0.397	1.322 ± 0.334	0.093 ± 0.075	4.667 ± 1.149
	XGB	0.972 ± 0.020	1.056 ± 0.437	0.767 ± 0.330	0.000 ± 0.001	2.650 ± 1.156
S4	MLP	0.951 ± 0.024	1.424 ± 0.341	1.025 ± 0.260	0.056 ± 0.080	3.575 ± 0.855
	RF	0.969 ± 0.030	1.070 ± 0.527	0.753 ± 0.415	0.000 ± 0.003	2.586 ± 1.387
	SVR	0.881 ± 0.024	2.280 ± 0.208	1.606 ± 0.182	0.148 ± 0.074	5.712 ± 0.622
	XGB	0.983 ± 0.015	0.813 ± 0.369	0.585 ± 0.274	−0.000 ± 0.000	2.018 ± 0.969
S5	MLP	0.962 ± 0.019	1.248 ± 0.338	0.895 ± 0.247	0.034 ± 0.064	3.049 ± 0.851
	RF	0.974 ± 0.030	0.964 ± 0.561	0.669 ± 0.431	−0.001 ± 0.004	2.256 ± 1.422
	SVR	0.893 ± 0.024	2.158 ± 0.236	1.504 ± 0.201	0.077 ± 0.059	5.304 ± 0.680
	XGB	0.992 ± 0.005	0.575 ± 0.220	0.404 ± 0.155	−0.000 ± 0.000	1.384 ± 0.527
S6	MLP	0.962 ± 0.016	1.261 ± 0.300	0.905 ± 0.215	0.006 ± 0.023	3.028 ± 0.638
	RF	0.983 ± 0.020	0.799 ± 0.379	0.554 ± 0.309	−0.002 ± 0.003	1.913 ± 1.101
	SVR	0.910 ± 0.012	1.985 ± 0.109	1.416 ± 0.087	0.165 ± 0.034	4.994 ± 0.272
	XGB	0.994 ± 0.006	0.444 ± 0.267	0.314 ± 0.187	0.000 ± 0.001	1.073 ± 0.636

Values are mean ± SD across eight outer LOPO iterations. In each iteration, training metrics were calculated on the complete outer-training dataset (seven production periods; n = 1,400 observations) after refitting the hyperparameter configuration selected in the inner loop. These values represent refit training performance and not inner-validation performance. R^2, coefficient of determination; RMSE, root mean square error; MAE, mean absolute error; MBE, mean bias error; MAPE, mean absolute percentage error; MLP, Multilayer Perceptron; RF, Random Forest; SVR, Support Vector Regression; XGB, Extreme Gradient Boosting.

Table S8. Outer-training refit performance of the four ML algorithms for predicting LPH under the six predictor scenarios (mean ± SD).

Scenario	Model	R^2	RMSE	MAE	MBE	MAPE
S1	MLP	0.920 ± 0.015	0.154 ± 0.012	0.113 ± 0.010	0.010 ± 0.022	1.543 ± 0.136
	RF	0.950 ± 0.026	0.117 ± 0.029	0.082 ± 0.025	−0.000 ± 0.000	1.118 ± 0.347
	SVR	0.904 ± 0.013	0.168 ± 0.012	0.119 ± 0.009	−0.003 ± 0.016	1.635 ± 0.115
	XGB	0.948 ± 0.010	0.123 ± 0.013	0.089 ± 0.011	0.000 ± 0.000	1.212 ± 0.150
S2	MLP	0.921 ± 0.019	0.151 ± 0.014	0.107 ± 0.012	−0.003 ± 0.013	1.470 ± 0.162
	RF	0.967 ± 0.022	0.095 ± 0.030	0.064 ± 0.026	−0.000 ± 0.000	0.875 ± 0.351
	SVR	0.907 ± 0.012	0.165 ± 0.012	0.114 ± 0.006	−0.008 ± 0.011	1.568 ± 0.086
	XGB	0.949 ± 0.010	0.121 ± 0.012	0.087 ± 0.010	−0.000 ± 0.000	1.186 ± 0.130
S3	MLP	0.927 ± 0.018	0.145 ± 0.018	0.106 ± 0.013	−0.005 ± 0.018	1.447 ± 0.174
	RF	0.977 ± 0.014	0.081 ± 0.023	0.053 ± 0.019	−0.000 ± 0.000	0.731 ± 0.263
	SVR	0.898 ± 0.024	0.172 ± 0.017	0.123 ± 0.024	−0.005 ± 0.008	1.688 ± 0.316
	XGB	0.964 ± 0.014	0.100 ± 0.021	0.071 ± 0.015	−0.000 ± 0.000	0.972 ± 0.207
S4	MLP	0.930 ± 0.015	0.143 ± 0.014	0.102 ± 0.012	−0.002 ± 0.012	1.399 ± 0.166
	RF	0.980 ± 0.012	0.073 ± 0.021	0.048 ± 0.018	−0.000 ± 0.000	0.661 ± 0.245
	SVR	0.917 ± 0.014	0.156 ± 0.009	0.104 ± 0.008	−0.006 ± 0.003	1.444 ± 0.103
	XGB	0.972 ± 0.014	0.088 ± 0.021	0.061 ± 0.015	0.000 ± 0.000	0.828 ± 0.205
S5	MLP	0.958 ± 0.010	0.110 ± 0.014	0.079 ± 0.010	0.010 ± 0.004	1.080 ± 0.139
	RF	0.991 ± 0.003	0.051 ± 0.007	0.031 ± 0.004	−0.000 ± 0.000	0.422 ± 0.060
	SVR	0.938 ± 0.008	0.135 ± 0.005	0.090 ± 0.014	−0.002 ± 0.008	1.241 ± 0.178
	XGB	0.982 ± 0.014	0.065 ± 0.033	0.045 ± 0.023	−0.000 ± 0.000	0.607 ± 0.314
S6	MLP	0.964 ± 0.013	0.103 ± 0.017	0.073 ± 0.012	−0.005 ± 0.014	0.996 ± 0.166
	RF	0.988 ± 0.003	0.058 ± 0.006	0.035 ± 0.004	0.000 ± 0.000	0.483 ± 0.056
	SVR	0.944 ± 0.012	0.127 ± 0.013	0.094 ± 0.023	−0.000 ± 0.008	1.283 ± 0.301
	XGB	0.987 ± 0.016	0.048 ± 0.038	0.034 ± 0.027	0.000 ± 0.000	0.458 ± 0.361

Values are mean ± SD across eight outer LOPO iterations. In each iteration, training metrics were calculated on the complete outer-training dataset (seven production periods; n = 1,400 observations) after refitting the hyperparameter configuration selected in the inner loop. These values represent refit training performance and not inner-validation performance. R^2, coefficient of determination; RMSE, root mean square error; MAE, mean absolute error; MBE, mean bias error; MAPE, mean absolute percentage error; MLP, Multilayer Perceptron; RF, Random Forest; SVR, Support Vector Regression; XGB, Extreme Gradient Boosting.
